# Study on inter-ethnic human differences in bioactivation and detoxification of estragole using physiologically based kinetic modeling

**DOI:** 10.1007/s00204-017-1941-x

**Published:** 2017-03-29

**Authors:** Jia Ning, Jochem Louisse, Bert Spenkelink, Sebastiaan Wesseling, Ivonne M. C. M. Rietjens

**Affiliations:** Division of Toxicology, Wageningen University, Stippeneng 4, 6708 WE Wageningen, The Netherlands

**Keywords:** Inter-ethnic difference, Chinese, Caucasian, Physiologically based kinetic modeling, Estragole

## Abstract

Considering the rapid developments in food safety in the past decade in China, it is of importance to obtain insight into what extent safety and risk assessments of chemicals performed for the Caucasian population apply to the Chinese population. The aim of the present study was to determine physiologically based kinetic (PBK) modeling-based predictions for differences between Chinese and Caucasians in terms of metabolic bioactivation and detoxification of the food-borne genotoxic carcinogen estragole. The PBK models were defined based on kinetic constants for hepatic metabolism derived from in vitro incubations using liver fractions of the two ethnic groups, and used to evaluate the inter-ethnic differences in metabolic activation and detoxification of estragole. The models predicted that at realistic dietary intake levels, only 0.02% of the dose was converted to the ultimate carcinogenic metabolite 1′-sulfooxyestragole in Chinese subjects, whereas this amounted to 0.09% of the dose in Caucasian subjects. Detoxification of 1′-hydroxyestragole, mainly via conversion to 1′-oxoestragole, was similar within the two ethnic groups. The 4.5-fold variation in formation of the ultimate carcinogenic metabolite of estragole accompanied by similar rates of detoxification may indicate a lower risk of estragole for the Chinese population at similar levels of exposure. The study provides a proof of principle for how PBK modeling can identify differences in ethnic sensitivity and provide a more refined risk assessment for a specific ethnic group for a compound of concern.

**Electronic supplementary material:**

The online version of this article (doi:10.1007/s00204-017-1941-x) contains supplementary material, which is available to authorized users.

## Introduction

Recently, the dose-dependent bioactivation and detoxification of estragole in different species, including human, has been studied by physiologically based kinetic (PBK) and dynamic (PBD) modeling (Punt et al. 2008, 2009, 2016). The models defined for the human population were specific for Caucasians, since the parameters used to describe the kinetics were derived using samples from relevant tissues from Caucasian origin. Considering the rapid developments in food safety in the past decade in China, it is of importance to obtain insight into to what extent safety and risk assessments of chemicals performed for the Caucasian population would also apply to the Chinese population. Given the fact that race diversity might result in the variability of dose-response relationships affecting the safety and efficacy of chemical exposures (Malinowski et al. 2008), the absence of knowledge in this field implies that harmonization in legislation on chemicals between regulatory bodies of Europe, USA and Asia is hampered. Several studies have shown ethnic differences in cytochrome P450 enzymes. Significant differences in reactions catalyzed by CYP1A2, CYP2C9, CYP2C19, and CYP2E1 in liver microsomes have been observed between Chinese and Caucasian samples (Yang et al. 2012). Barter et al. (2013) developed PBK models and used them for in vitro to in vivo extrapolation to predict the P450-mediated pharmacokinetics of selected drugs in the Chinese population. The results showed that the predicted clearances for phenacetin, tolbutamide, desipramine, omeprazole, alprazolam (intravenous), alprazolam (oral), midazolam (intravenous), and midazolam (oral) in Chinese subjects were predicted to be 36, 25, 43, 51, 21, 22, 24, and 17% lower, respectively, than in Caucasian subjects, and the experimentally observed clearances in treated Chinese individuals were 28, 2, 42, 75, 20, 21, 19, and 62% lower, respectively, than in Caucasian volunteers (Barter et al. 2013).

**Fig. 1:**
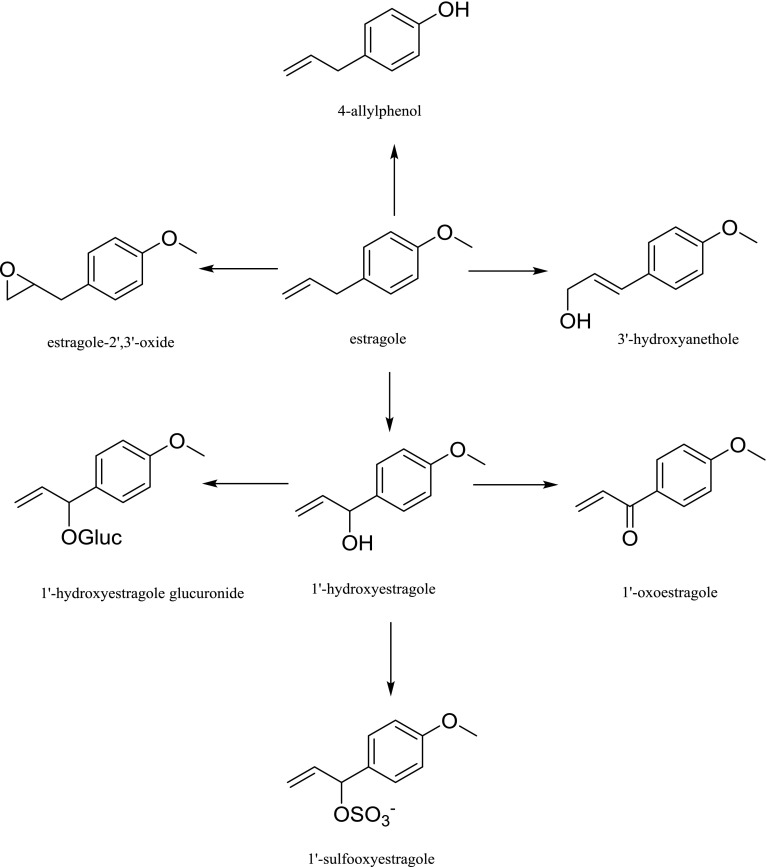
Metabolic pathways of estragole

Thus, inter-ethnic differences in metabolism and metabolic bioactivation and detoxification may occur. However, systematic attempts to predict the kinetic processes and related toxicity in different populations using physiologically based kinetic (PBK) modeling are lacking. The aim of the present study was to determine PBK modeling-based predictions for differences between Chinese and Caucasians in terms of metabolic bioactivation and detoxification of the food-borne genotoxic carcinogen estragole (Fig. 1). Estragole (1-allyl-4-methoxybenzene) is an alkenylbenzene that is naturally present in a variety of herbs and spices, such as fennel, basil, and tarragon (Smith et al. 2002). Consumption of herbs, spices, and their essential oils and food products containing these is an important route of exposure to estragole. The Flavor and Extract Manufacturers Association (FEMA) estimated the daily intake of estragole to be less than 0.01 mg/kg bw/day based on the annual production volume data of estragole for use in flavorings (Smith et al. 2002). Estragole is known to be genotoxic and carcinogenic in rodents at high-dose levels (Drinkwater et al. 1976; Miller et al. 1983). Bioactivation of estragole to a DNA reactive ultimate carcinogen proceeds by cytochrome P450 mediated conversion to 1′-hydroxyestragole and subsequent conversion of 1′-hydroxyestragole to the ultimate carcinogen 1′-sulfooxyestragole by sulfotransferases (SULTs) (Fig. 1). Detoxification of 1′-hydroxyestragole proceeds by glucuronidation to 1′-hydroxyestragole glucuronide and oxidation to 1′-oxoestragole (Fig. 1). Given the variety of biotransformation enzymes involved in estragole bioactivation and detoxification, estragole was selected as an adequate model compound to study ethnic differences in bioactivation and detoxification. To define the PBK models for the Chinese and Caucasian populations, kinetic constants for the various biotransformation reactions of estragole were quantified using in vitro incubations with relevant tissue fractions of the two ethnic groups. The outcomes predicted by the PBK model for the Chinese population were compared to those predicted by the PBK model for Caucasians to evaluate the inter-ethnic differences in metabolic activation and detoxification of estragole and to demonstrate the potential of PBK modeling to study such inter-ethnic variability.

## Materials and methods

### Chemicals and biological materials

Estragole (1-allyl-4-methoxybenzene), dimethylsulfoxide (DMSO), alamethicin, uridine 5′-diphosphoglucuronic acid (UDPGA), 3′-phosphoadenosine-5′-phosphosulfate (PAPS), phenacetin, acetaminophen, coumarin, 7-hydroxycoumarin, 7-hydroxycoumarin sulfate, glucose-6-phosphate dehydrogenase, and reduced L-glutathione (GSH) were purchased from Sigma–Aldrich (Steinheim, Germany). Potassium dihydrogen phosphate, dipotassium hydrogen phosphate trihydrate, hydrochloric acid (37%), trifluoroacetic acid (TFA), and magnesium chloride were purchased from VWR International (Darmstadt, Germany). Reduced nicotinamide adenine dinucleotide phosphate (NADPH), nicotinamide adenine dinucleotide phosphate nicotinamide (NADP^+^), adenine dinucleotide (NAD^+^), and glucose-6-phosphate were obtained from Roche Diagnostics (Mannheim, Germany). Acetonitrile (UPLC/MS grade) was obtained from Biosolve BV (Valkenswaard, Netherlands). 1′-Hydroxyestragole, 4-allylphenol, estragole-2′,3′-oxide, 3′-hydroxyanethole, and 1′-oxoestragole were synthesized as previously described by Punt et al. (2007, 2008). Chinese liver microsomes and Chinese S9, made from 40 donors, were purchased from PrimeTox (Wuhan, China). Caucasian liver microsomes were purchased from BD Gentest (Woburn, MA, USA), and Caucasian liver S9 was purchased from Corning (Amsterdam, Netherlands). All microsomes and S9 were of pooled mixed gender.

### In vitro incubations

#### Assessment of metabolic capabilities of CYP and SULT enzymes in Chinese and Caucasian liver samples

The quality of the Chinese and Caucasian liver microsomes was checked by measuring the activity of CYP1A2 and CYP2A6 by the method of Yang et al. (2012). The quality of Chinese and Caucasian liver S9 samples was checked by measuring the SULT activity based on the method provided by Wang et al. (2006). The details of the methods for the microsomal and S9 in vitro incubations and UPLC analysis for assessing the metabolic capabilities of CYP and SULT enzymes can be found in the supporting materials 1.

#### Microsomal metabolism of estragole

The kinetic constants for the microsomal conversion of estragole were determined as previously described by Punt et al. (2009). Briefly, mixed gender Chinese or Caucasian liver microsomes were incubated with estragole in the presence of NADPH. The incubation mixtures contained (final concentrations) 3 mM NADPH and 1 mg/mL microsomal protein in 0.2 M Tris–HCl (pH 7.4). Incubations were performed for 10 min at substrate concentrations ranging from 25 to 1000 μM, after which the reaction was terminated by adding 25 μL ice-cold acetonitrile. Blank incubations were performed in the absence of the cofactor NADPH. All incubations were performed in triplicate. In the supporting materials 2, detailed information can be found (Table S1 can be used to keep track of detailed information).

#### Glucuronidation of 1′-hydroxyestragole

Pooled mixed gender Chinese or Caucasian liver microsomes were incubated with 1′-hydroxyestragole in the presence of UDPGA. As previously described by Punt et al. (2009), the incubation mixtures contained (final concentrations) 10 mM UDPGA, and 1 mg/mL microsomal protein in 0.2 M Tris–HCl (pH 7.4) with 10 mM MgCl_2_. Incubations were carried out for 6 h, and the reaction was terminated by adding 25 μL ice-cold acetonitrile. Blank incubations were performed in the absence of the cofactor UDPGA. All incubations were performed in triplicate. In the supporting materials 2, detailed information can be found (Table S1 can be used to keep track of detailed information).

#### Oxidation of 1′-hydroxyestragole

Mixed gender Chinese or Caucasian liver S9 was incubated with 1′-hydroxyestragole in the presence of NAD^+^ and GSH, the latter added to trap the transient 1′-oxoestragole. Formation of the 1′-oxoestragole adducts with GSH forming GS-1′-oxoestragole reflects the formation of 1′-oxoestragole (Punt et al. 2009). The incubations had a final volume of 100 μL, containing (final concentrations) 3 mM NAD^+^, 2 mM GSH, and 1 mg/mL liver S9 in 0.2 M Tris–HCl (pH 7.4), as described previously by Punt et al. (2016). The reactions were terminated after 10 min by the addition of 25 μL ice-cold acetonitrile. Blank incubations were performed without cofactor NAD^+^. All incubations were performed in triplicate. In the supporting materials 2, detailed information can be found (Table S1 can be used to keep track of detailed information).

#### Sulfation of 1′-hydroxyestragole

The formation of 1′-sulfooxyestragole was determined by incubating 0.2 mg/mL pooled mixed gender Chinese or Caucasian liver S9 in the presence of 0.2 mM PAPS as cofactor and 10 mM GSH as trapping agent for the reactive 1′-sulfooxyestragole in 0.1 M potassium phosphate (pH 8.0). The incubations were carried out for 2 h, and the reactions were terminated by adding 25 μL ice-cold acetonitrile. The blank samples were performed without cofactor. All incubations were performed in triplicate. In the supporting materials 2, detailed information can be found (Table S1 can be used to keep track of detailed information).

### UPLC analysis

#### UPLC analysis of estragole metabolites

Before UPLC analysis, all samples were centrifuged for 5 min at 16,000 g to precipitate microsomal proteins. Supernatant of each sample was analyzed on UPLC using a BEH C18 (1.7 μm, 2.1 × 50 mm) column with a guard column and a diode array detector (Acquity, Waters). The gradient for analysis of metabolites of estragole can be found in supporting materials 2. Table S1 can be used to keep track of detailed information. Identification of microsomal metabolites of estragole, including 4-allylphenol, estragole-2′,3′-diol, 1′-hydroxyestragole, and 3′-hydroxyanethole, and M5 was achieved by comparison of the UV spectra and retention times of formed metabolites to those of synthesized reference compounds identified previously (Punt et al. 2007, 2008). Formation of 4-allylphenol, estragole-2′,3′-diol, and 1′-hydroxyestragole was quantified by comparing the peak areas to those of the corresponding reference standard curves at wavelength 225 nm (Agharahimi and LeBel 1995; Drinkwater et al. 1976; Iyer et al. 2003; Luo et al. 1992). Because the UV spectrum of M5 is similar to that of estragole-2′,3′-diol, quantification of M5 could be achieved by comparison of the peak area to the calibration curve of estragole-2′,3′-diol at 225 nm. 3′-Hydroxyanethole was quantified by comparison of the peak areas of the metabolite in the chromatograms obtained at a wavelength of 206 nm to the calibration curve of the synthesized reference compound (Agharahimi and LeBel 1995; Drinkwater et al. 1976; Iyer et al. 2003; Luo et al. 1992). The amounts of formed microsomal estragole metabolites were corrected for the amounts detected in the blank incubations performed without the respective cofactor NADPH.

#### UPLC analysis of 1′-hydroxyestragole metabolites

Before UPLC analysis, all samples were centrifuged for 5 min at 16, 000 g to precipitate microsomal or cytosolic proteins. Supernatant of each sample was analyzed on UPLC using a BEH C18 (1.7 μm, 2.1 × 50 mm) column with a guard column and a diode array detector (Acquity, Waters). Secondary metabolism of 1′-hydroxyestragole includes glucuronidation, oxidation, and sulfation. The gradient of analysis of the metabolites of 1′-hydroxyestragole can be found in supporting materials 2. Table S1 can be used to track more detailed information.

Identification of 1′-hydroxyestragole glucuronide was achieved by the fact that it was the only metabolite formed and by LC-MS analysis as reported previously (Punt et al. 2008). Both 1′-hydroxyestragole glucuronide and 1′-hydroxyestragole have the same UV spectrum and the same extinction coefficient at a wavelength of 225 nm. Thus, the quantification of 1′-hydroxyestragole glucuronide could be achieved by comparing the peak area to the calibration curve of 1′-hydroxyestragole at wavelength 225 nm (Punt et al. 2009). The amounts of 1′-hydroxyestragole glucuronide formed were corrected for the amounts detected in the blank incubations performed without the respective cofactor UDPGA.

Identification of the GS-1′-oxoestragole which reflects the formation of 1′-oxoestragole was done by comparing the UV spectra and retention time of the formed GSH adduct to those of GS-1′-oxoestragole identified as described previously (Punt et al. 2009). Quantification of the GSH conjugate of 1′-oxoestragole was achieved by comparing the peak area to the calibration curve of GS-1′-oxoestragole at a wavelength of 280 nm prepared as described previously by Punt et al. (2009, 2016). Briefly, the calibration curve of GS-1′-oxoestragole was prepared by incubating 40 μM 1′-oxoestragole with a range of GSH concentrations. The reactions were incubated for 4 h after which maximal formation of GS-1′-oxoestragole was previously shown to be reached (Punt et al. 2009). The amounts of 1′-oxoestragole formed were corrected for the amounts detected in the blank incubations performed without the respective cofactor NAD^+^.

Since the UV spectrum of 3′-hydroxyanethole is similar to that of the GSH adduct of 1′-sulfooxyestragole, quantification of 1′-sulfooxyestragole could be achieved by comparing the peak area of the GSH adduct of 1′-sulfooxyestragole to the calibration curve of 3′-hydroxyanethole at a wavelength 260 nm as described for the quantification of 1′-sulfooxysafrole and 1′-sulfooxyelemicin (Martati et al. 2012; Van den Berg et al. 2012). The amount of 1′-sulfooxyestragole GSH adduct formed was corrected for the amount detected in the blank samples performed without the respective cofactor PAPS.

### Kinetic analysis

The data for the formation of estragole and 1′-hydroxyestragole metabolites with increasing substrate concentration [*S*] were fitted to the standard Michaelis–Menten equation:

$$\upsilon = {V_{{\text{max}}}}/(1 + ({K_{\text{m}}}/\left[ S \right]).$$

The apparent maximum velocity (*V*_max_) and the apparent Michaelis–Menten constant (*K*_m_) were determined by fitting the data to this equation using GraphPad Prism version 5.0 (GraphPad software, San Diego California USA).

### PBK model structure

**Fig. 2:**
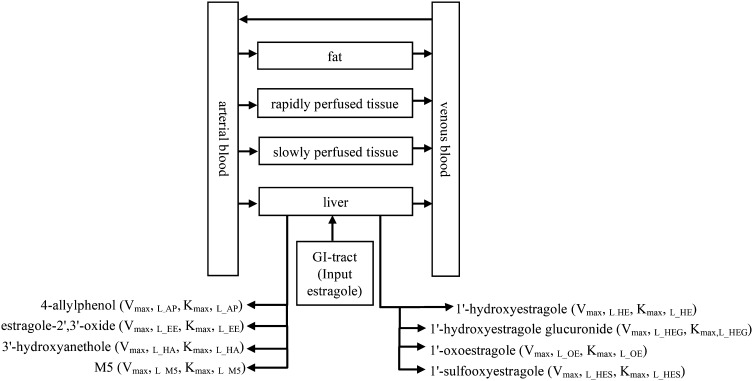
Schematic diagram of the PBK model for estragole in human, including the name of kinetic parameters for metabolites of estragole and 1′-hydroxyestragole as used in the mass balance equations of the PBK model

The PBK models developed in this study were based on the PBK models previously defined by Punt et al. (2008). The models have six compartments, including blood, fat, rapidly perfused tissue, slowly perfused tissue, liver, and GI tract that are mutually connected through the systemic circulation. A schematic diagram of the PBK models for estragole kinetics is presented in Fig. 2, and the code of the models can be found in the supporting materials 6. Table 1 summarizes the physiological parameters for Caucasian and Chinese subjects, respectively, which were derived from the literature (Brown et al. 1997; NHFPC 2007, 2014b). Based on the method described by DeJongh et al. (1997; Punt et al. 2016), partition coefficients were estimated based on the log *K*_ow_. The log *K*_ow_ values for estragole and 1′-hydroxyestragole were estimated by ChemBio 3D 2010 (CambrigeSoft, USA). Model equations were coded and numerically integrated in Berkely Madonna (Macey and Oster, UC Berkeley, CA, USA) using the Rosenbrock’s algorithm for stiff systems.

**Table 1:** Parameters used in the physiologically based kinetic model for estragole in Chinese and Caucasian populations as obtained from the literature

Model parameters	Chinese^a,c^	Caucasian^b,c^
Physiological parameters		
Body weight (kg)	60	70
Percentage of body weight		
Liver	2.3	2.6
Fat	18.7	21.4
Rapidly perfused	5.3	5
Slowly perfused	54.3	51.7
Blood	7.9	7.9
Cardiac output (l/hr/kg bw^0.74^)	15	15
Percentage of cardiac output		
Liver	26.3	22.7
Fat	6.75	5.2
Rapidly perfused	43.8	47.3
Slowly perfused	23.3	24.8
Tissue: blood partition coefficients		
Estragole		
Liver	6.5	6.5
Fat	105	105
Rapidly perfused	6.5	6.5
Slowly perfused	4.1	4.1
1′-Hydroxyestragole		
Liver	1.6	1.6

^a^NHFPC (2007); NHFPC (2014b)

^b^Brown et al. (1997)

^c^DeJongh et al. (1997)

Estragole was assumed to directly enter from the gastrointestinal tract via the portal vein into the liver with an absorption rate constant (*K*_a_) of 1.0 h^−1^ as a first-order process. This value was used based on the fact that complete and fast absorption of estragole from the GI tract has been observed (Anthony et al. 1987). As reported by Punt et al. (2009), conversion of estragole mainly occurs in the liver and not in other organs. In addition, since the liver is known to be the major target organ for estragole-induced tumor formation in rat and mice, the PBK model focused on metabolism of estragole and 1′-hydroxyestragole in the liver. 1′-Hydroxyestragole, 2′,3′-estragole oxide, 3′-hydroxyanethole, 4-allylphenol, and an unidentified minor metabolite referred to as M5 were formed in incubations with liver microsomes from both populations (see “Results” section), and conversions of estragole into these metabolites were included in the liver compartment of the models. In both models, descriptions of the conversions of 1′-hydroxyestragole into its further metabolites were included, but the conversions of 2′,3′-estragole oxide, 3′-hydroxyanethole, 4-allylphenol, and M5 to their further metabolites were not included, since these are assumed to not influence the formation of the ultimate carcinogenic metabolite 1′-sulfooxyestragole. The mass balance equations for microsomal conversion of estragole when using Chinese or Caucasian liver microsomes were described as follows:

$$\begin{gathered} {\text{dAL}_\text{E}}/dt{\text{ }} = {\text{ }}dUptake_E/dt \hfill \\ \quad \quad \quad \quad + {\text{ }}{\text{QL}}{\text{ }} \times {\text{ }}({\text {CA}_\text{E}}{\text{ }} - {\text{ }}{\text{CL}_\text{E}}/ {\text{PL}_\text{E}}) \hfill \\ \quad \quad \quad \quad {\text{ }} - {V_{\max ,{\text{ }}{\text {L}\_\text{AP}}}}{\text{ }} \times {\text{ }}({\text{CL}_\text{E}}/ {\text{PL}_\text{E}})/({K_{m,{\text{ }}{\text {L}\_\text{AP}}}}{\text{ }} + {\text{ }}{\text{CL}_\text{E}}/ {\text{PL}_\text{E}}) \hfill \\ \quad \quad \quad \quad {\text{ }} - {V_{\max ,{\text{ }}{\text {L}\_\text{HE}}}}{\text{ }} \times {\text{ }}({\text{CL}_\text{E}}/ {\text{PL}_\text{E}})/({K_{m,{\text{ }}{\text {L}\_\text{HE}}}}{\text{ }} + {\text{ }}{\text{CL}_\text{E}}/ {\text{PL}_\text{E}}) \hfill \\ \quad \quad \quad \quad {\text{ }} - {V_{\max ,{\text{ }}{\text {L}\_\text{EE}}}}{\text{ }} \times {\text{ }}({\text{CL}_\text{E}}/ {\text{PL}_\text{E}})/({K_{m,{\text{ }}{\text {L}\_\text{EE}}}} + {\text{ }}{\text{CL}_\text{E}}/ {\text{PL}_\text{E}}) \hfill \\ \quad \quad \quad \quad \, - {V_{\max ,{\text{ }}{\text {L}\_\text{HA}}}}{\text{ }} \times {\text{ }}({\text{CL}_\text{E}}/ {\text{PL}_\text{E}})/({K_{m,{\text{ }}{\text {L}\_\text{HA}}}} + {\text{ }}{\text{CL}_\text{E}}/ {\text{PL}_\text{E}}) \hfill \\ \quad \quad \quad \quad \, - {V_{\max ,{\text{ }}{\text {L}\_\text{M5}}}}{\text{ }} \times {\text{ }}({\text{CL}_\text{E}}/ {\text{PL}_\text{E}})/({K_{m,{\text{ }}{\text {L}\_\text{M5}}}} + {\text{ }}{\text{CL}_\text{E}}/ {\text{PL}_\text{E}}) \hfill \\ dUptak{e_E}/dt{\text{ }} = {\text{ }} - {\text{dAGI}_\text{E}}/dt{\text{ }} = {\text{ }}Ka{\text{ }} \times {\text{ }}{\text{AGI}_\text{E}},{\text{ }}{\text{AGI}_\text{E}}(0){\text{ }} = {\text{ }}oral{\text{ }}dose \hfill \\ {\text{CL}_\text{E}} = {\text{ }}{\text{AL}_\text{E}}/{\text{VL}} \hfill \\ \end{gathered}$$

where AL_E_ is the amount of estragole in the liver tissue (μmol). Uptake_E_ is the amount of estragole taken up from the GI tract (μmol), QL is the blood flow rate to and from the liver tissue (L/h), CA_E_ is the estragole concentration in the arterial blood (μmol/L), and CL_E_ is the estragole concentration in the liver tissue. PL_E_ is the liver/blood partition coefficient, *V*_max, L_M_ and *K*_m, L_M_ are the maximum rate of formation and Michaelis–Menten constant for the metabolites 4-allylphenol (AP), 1′-hydroxyestragole (HE), estragole-2′,3′-oxide (EE), 3′-hydroxyanethole (HA) and metabolite 5 (M5) in the liver tissue, AGI_E_ (μmol) is the amount of estragole in the GI tract, and VL is the volume of the liver. The mass balance equation for the metabolism of 1′-hydroxyestragole in the liver was as follows:

$$ \begin{aligned} {\text{dAL}}_{\rm HE} /d{\text{t}} & = V_{{{\text{max, L}\_{\text{HE}}}}} \times ({\text{CL}}_{E} /{\text{PL}}_{E} )/(K_{{\text {m, L}}\_{\text{HE}}}+ {\text{CL}}_{E} /{\text{PL}}_{E} ) \\ & \quad - V_{{\max , {\text {L}}\_{\text{HEG}}}} \times ({\text{CL}}_{{{\text{HE}}}} /{\text{PL}}_{{{\text{HE}}}} )/(K_{{m,{\text {L}}\_{\text{HEG}}}} + {\text{CL}}_{{{\text{HE}}}} /{\text{PL}}_{{{\text{HE}}}} ) \\ & \quad - V_{{\max , {\text {L}}\_{\text{OE}}}} \times({\text{CL}}_{{{\text{HE}}}} /{\text{PL}}_{{{\text{HE}}}} )/(K_{{m,{\text {L}}\_{\text{OE}}}} + {\text{CL}}_{{{\text{HE}}}} / {\text{PL}}_{{{\text{HE}}}} ) \\ & \quad - V_{{\max ,{\text {L}}\_{\text{HES}}}} \times ({\text{CL}}_{{{\text{HE}}}} /{\text{PL}}_{{{\text{HE}}}} )/(K_{{m,{\text{L}}\_{\text{HES}}}} + {\text{CL}}_{{{\text{HE}}}} / {\text{PL}}_{{{\text{HE}}}} )\\ {\text{CL}}_{{{\text{HE}}}} & = {\text{ AL}}_{{{\text{HE}}}}/ {\text{VL}} \\ \end{aligned} $$

where AL_HE_ is the amount of 1′-hydroxyestragole in the liver tissue (μmol), CL_HE_ is the 1′-hydroxyestragole concentration in the liver tissue (μmol/L), PL_HE_ is the liver/blood partition coefficient of 1′-hydroxyestragole, *V*_max, L_M,_ and *K*_m, L_M_ are the maximum rate and the Michaelis–Menten constant for the formation of 1′-hydroxyestragole glucuronide (HEG), 1′-oxoestragole (OE) and 1′-sulfooxyestragole (HES) in the liver tissue.

The kinetic constants for metabolites formed were determined in vitro in the present study. *V*_max_ values expressed as nmol/min/(mg microsomal or S9 protein) were scaled to the *V*_max_ per μmol/h/(g liver) using mirosomal and S9 protein yields of 35 and 143 mg/g liver, respectively, as previously described by Punt et al. (2009), Al-Subeihi et al. (2012), Martati et al. (2012), and Van den Berg et al. (2012). Currently, no data are available specifically for the liver microsomal and S9 protein relevant for the Chinese tissue samples. Therefore, the value of liver microsomal protein and liver S9 protein available for the Caucasians was used for the Chinese population.

### Sensitivity analysis

To identify which parameters have the greatest impact on the model predictions on the formation of 1′-hydroxyestragole and 1′-sulfooxyestragole, a sensitivity analysis was performed. Normalized sensitivity coefficients (SC) were determined using the following equation:

$${\text{SC}}\; = \;\left( {C^{\prime} - C} \right)/\left( {P^{\prime} - P} \right)\; \times \;(P/C)$$

where *C* is the initial value of the model output; *C*′ is the modified model output resulting from a 5% increase of the parameter value; *P* is the initial parameter value; and *P*′ is the modified parameter value (Evans and Andersen 2000). A 5% increase in parameter values was chosen to analyze the effect of a change in parameter values on formation of 1′-hydroxyestragole and 1′-sulfooxyestragole at a dose 0.01, 5, and 150 mg/kg bw/day for 24 h exposure, representing respectively a realistic daily intake (Smith et al. 2002), an intake that may result from supplement use (Van Den Berg et al. 2011) and a dose level known to cause liver tumors in rodent bioassays (Drinkwater et al. 1976; Miller et al. 1983). Each parameter was analyzed individually, while other parameters were kept as their initial value.

### Comparison of the PBK model-based predictions for bioactivation and detoxification of estragole in the Chinese and Caucasian population

The PBK model-based predictions for the formation of metabolites of estragole and 1′-hydroxyestragole in the Chinese population were compared with the predicted formation of these metabolites in the Caucasian population. Model predictions were made for a period of 24 h after exposure.

## Results

### Metabolic capabilities of CYP and SULT enzymes

The results of metabolic capabilities of CYP and SULT enzymes can be found in supporting materials 3. The quality of the Chinese tissue samples used to determine the various kinetic parameters appeared to be in line with what has been reported before (Yang et al. 2012; Wang et al. 2006).

### Microsomal conversion of estragole

The microsomal conversion of estragole was determined by incubating estragole with Chinese or Caucasian liver microsomes in the presence of NADPH. Figure S1 in supporting materials 4 presents a representative chromatogram of an incubation with Chinese liver microsomes showing the formation of estragole-2′,3′-diol (Rt = 2.9 min), 1′-hydroxyestragole (Rt = 4.7 min), 3′-hydroxyanethole (Rt = 4.9 min), M5 (Rt = 5 min) and 4-allylphenol (Rt = 6.8 min). Since the metabolite referred to as M5 was only formed in a relatively small amount in incubations with liver microsomes from both ethnic groups, its identification was not deemed essential. Due to the presence of epoxide hydrolase in the human liver microsomes, the formation of estragole-2′,3′-diol reflects formation of estragole-2′,3′-oxide (Guenthner and Luo 2001; Luo and Guenthner 1996; Luo et al. 1992).

**Fig. 3:**
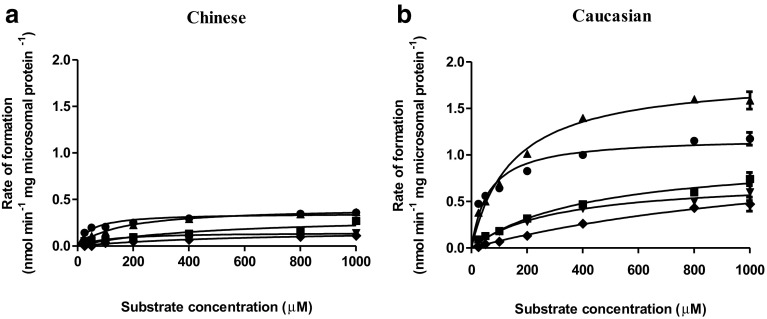
Concentration-dependent rate of metabolic conversion of estragole in incubations with Chinese (**a**) or Caucasian (**b**) liver microsomes. *Data points* represent mean values ± SEM of three individual experiments for each metabolite, including estragole-2′,3′-oxide (*closed upward triangle*), 1′-hydroxyestragole (*closed circle*), 3′-hydroxyanethole (*closed square*), 4-allylphenol (*closed downward triangle*), and M5 (*closed diamond*)

The estragole concentration-dependent rates of formation of estragole-2′,3′-oxide, 1′-hydroxyestragole, 3′-hydroxyanethole, M5, and 4-allylphenol by Chinese or Caucasian liver microsomes is shown in Fig. 3. The kinetic constants for these metabolic conversions of estragole and the catalytic efficiency, calculated as *V*_max_/*K*_m_, obtained from these data are summarized in Table 2.

**Table 2:** Kinetic constants (average ± SEM) for metabolic conversion of estragole and 1′-hydroxyestragole in incubations with Chinese or Caucasian liver tissue fractions

Ethnic group	Chinese			Caucasian		
Metabolite	*K*_m_ (µM)	*V*_max_ (nmol /min/mg microsomal or S9 protein)	Catalytic efficiency^a^ (µL/min/mg microsomal or S9 protein)	*K*_m_ (µM)	*V*_max_ (nmol /min/mg microsomal or S9 protein)	Catalytic efficiency^a^ (µL/min/mg microsomal or S9 protein)
Conversion of estragole						
4-Allylphenol	115 ± 24	0.15 ± 0.01	1.3	308 ± 71	0.74 ± 0.07	2.4
Estragole-2′,3′-oxide	161 ± 17	0.42 ± 0.01	2.6	146 ± 15	1.85 ± 0.06	12.7
1′-Hydroxyestragole	49 ± 8	0.35 ± 0.01	7.9	62 ± 9	1.19 ± 0.04	19.3
3′-Hydroxyanethole	450 ± 219	0.32 ± 0.07	0.71	429 ± 80	0.99 ± 0.08	2.3
M5	618 ± 164	0.18 ± 0.02	0.29	1451 ± 615	1.18 ± 0.34	0.82
Conversion of 1′-hydroxyestragole						
1′-Hydroxyestragole glucuronide	4656 ± 1247	1.63 ± 0.33	0.35	4607 ± 878	4.29 ± 0.61	0.93
1′-Oxoestragole	403 ± 137	1.82 ± 0.28	4.5	521 ± 265	2.8 ± 0.70	5.4
1′-Sulfooxyestragole	694 ± 321	0.0014 ± 0.0002	0.0021	423 ± 159	0.004 ± 0.0005	0.01

^a^ *V*_max_/*K*_m_ × 1000

The results showed that estragole-2′,3′-oxide was the most abundant metabolite formed in incubations with liver microsomes from both populations, followed by 1′-hydroxyestragole. Analysis of catalytic efficiencies for the formation of the microsomal estragole metabolites revealed that the catalytic efficiency for formation of 1′-hydroxyestragole was the highest, followed by the formation of estragole-2′,3′-oxide, 4-allylphenol, 3′-hydroxyanethole and M5 in both populations. Thus, formation of 1′-hydroxyestragole was the major microsomal metabolic pathway of estragole, whereas formation of M5 was the least important route of estragole metabolism. The catalytic efficiency for the formation of 1′-hydroxyestragole was higher than that for the other microsomal metabolites because of a relatively low *K*_m_. Comparison of the catalytic efficiencies obtained for the two ethnic groups revealed that the catalytic efficiencies for the formation of 4-allylphenol, estragole-2′,3′-oxide, 1′-hydroxyestragole, 3′-hydroxyanethole, and M5 in incubations with the Chinese samples were 1.8-, 4.9-, 2.4-, 3.2-, and 2.9-fold lower, respectively, than those in incubations with Caucasian samples. The relatively low catalytic efficiencies for the Chinese samples were mainly due to relatively low *V*_max_ values, which were 5.0-, 4.4-, 3.4-, 3.1-, and 6.5-fold lower than the *V*_max_ for *O*-demethylation, epoxidation, 1′-hydroxylation, 3′-hydroxylation, and the reaction for the formation of M5 in Caucasian samples, respectively. The apparent *K*_m_ values for these reactions by Chinese and Caucasian samples were similar.

### Glucuronidation of 1′-hydroxyestragole

Kinetic constants for the formation of 1′-hydroxyestragole glucuronide were determined by incubations with Chinese or Caucasian liver microsomes in the presence of UDPGA and 1′-hydroxyestragole. 1′-Hydroxyestragole glucuronide eluted at 2.6 min and was identified by LC-MS as previously described (Punt et al. 2008). The metabolite was quantified using the calibration curve of 1′-hydroxyestragole on the basis of the similarity in their UV spectra assuming a similar molar extinction coefficient.

**Fig. 4:**
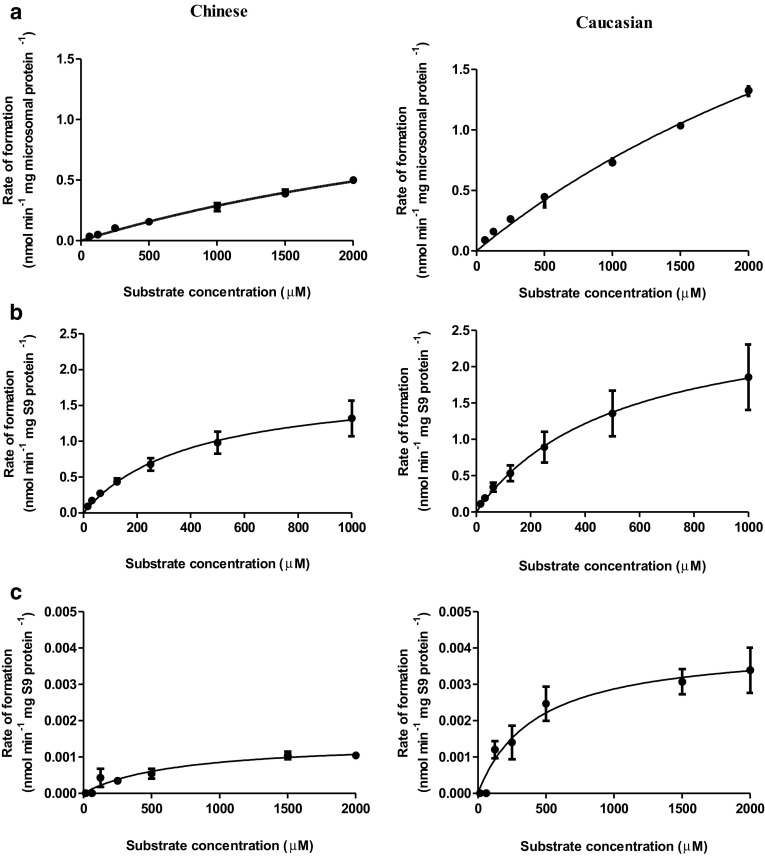
Concentration-dependent rate of metabolic conversion of 1′-hydroxyestragole to (**a**) 1′-hydroxyestragole glucuronide, (**b**) 1′-oxoestragole, and (**c**) 1′-sulfooxyestragole in incubations with Chinese or Caucasian liver tissue fractions. *Data points* represent mean values ± SEM of three individual experiments for each metabolite

Figure 4a shows the 1′-hydroxyestragole concentration-dependent rate of formation of 1′-hydroxyestragole glucuronide by liver microsomes and kinetic constants derived from these plots are displayed in Table 2. The apparent *K*_m_ and *V*_max_ for formation of 1′-hydroxyestragole glucuronide by Chinese samples were 4656 µM and 1.63 nmol/min/(mg microsomal protein), respectively, whereas *K*_m_ and *V*_max_ for formation of 1′-hydroxyestragole glucuronide by Caucasian samples were 4607 µM and 4.29 nmol/min/(mg microsomal protein), respectively. These values result in a catalytic efficiency that is 2.7-fold lower for Chinese than for Caucasian subjects.

### Oxidation of 1′-hydroxyestragole

1′-Oxoestragole was formed in incubations with 1′-hydroxyestragole and Chinese or Caucasian liver S9 using NAD^+^ as cofactor and GSH to trap 1′-oxoestragole forming GS-1′-oxoestragole. Chromatographic analysis revealed that GS-1′-oxoestragole eluted at 2.6 min based on its previous identification (Punt et al. 2009).

The rate of oxidation of 1′-hydroxyestragole with increasing concentration of 1′-hydroxyestragole is shown in Fig. 4b and Table 2 displays the kinetic constants for formation of 1′-oxoestragole derived from these data. The apparent *K*_m_ for formation of 1′-oxoestragole by Chinese samples was 403 µM, and the *V*_max_ was 1.82 nmol/min/(mg S9 protein). For Caucasian samples, the apparent *K*_m_ and the *V*_max_ were 521 µM and 2.8 nmol/min/(mg S9 protein), respectively. The catalytic efficiencies for formation of 1′-oxoestragole by both ethnic groups were similar.

### Sulfation of 1′-hydroxyestragole

Formation of the ultimate carcinogenic metabolite 1′-sulfooxyestragole upon sulfation of 1′-hydroxyestragole was observed in incubations with Chinese as well as Caucasian liver S9 in the presence of PAPS and GSH. GSH was used to trap the transient 1′-sulfooxyestragole forming the GSH adduct of 1′-sulfooxyestragole. Chromatographic analysis revealed a peak at 1.4 min, identified as the GSH adduct of 1′-sulfooxyestragole. This identification was based on the fact that the peak was absent in the incubations with PAPS and S9 in the absence of GSH (chromatogram not shown), and the previous identification of this (Punt et al. 2007) and similar alkenylbenzene 1′-sulfooxy adducts by LC-MS (Al-Malahmeh et al. 2017; Al-Subeihi et al. 2012; Alajlouni et al. 2016; Martati et al. 2012).

Figure 4c presents the 1′-hydroxyestragole concentration-dependent rate of formation of 1′-sulfooxyestragole in incubations with Chinese or Caucasian liver S9 as determined by quantification of the GSH adduct of 1′-sulfooxyestragole. Table 2 presents a summary of the kinetic constants for formation of 1′-sulfooxyestragole in the two ethnic groups derived from these data. The catalytic efficiency for formation of 1′-sulfooxyestragole in incubation with Chinese samples was fivefold lower than those in incubations with Caucasian samples.

### Comparison of the kinetic constants for conversion of estragole and 1′-hydroxyestragole by Chinese and Caucasian liver tissue fractions

Table 3 displays the scaled kinetic parameters for conversion of estragole and 1′-hydroxyestragole by Chinese or Caucasian liver tissue fractions. In this table, *V*_max_ values that were obtained in vitro expressed as nmol/min/(mg microsomal protein or S9) (Table 2) were scaled to whole liver and expressed in µmol/h/(g tissue). A scaled catalytic efficiency (*V*_max_ in vivo/*K*_m_) for formation of the different metabolites of estragole and 1′-hydroxyestragole could be calculated. The catalytic efficiencies thus obtained reveal that formation of the proximate carcinogenic metabolite of estragole, 1′-hydroxyestragole, was 2.5-fold lower for Chinese liver microsomes as compared to Caucasian liver micorosomes. This difference in catalytic efficiency was mainly due to the low *V*_max_ in Chinese liver microsomes, since the value of *V*_max_ in Chinese liver incubations was 3.5-fold lower than in Caucasian liver incubations. Epoxidation of estragole turned out to be the main route of microsomal metabolism of estragole in both ethnic groups, although the catalytic efficiency for formation of estragole-2′,3′-oxide was fivefold lower for the Chinese population. This lower catalytic efficiency was mainly due to a fourfold lower *V*_max_ for Chinese liver samples.

**Table 3:** Scaled kinetic constants (average ± SEM) for metabolic conversion of estragole and 1′-hydroxyestragole from incubations with Chinese or Caucasian liver tissue fractions

Ethnic	Chinese			Caucasian		
Metabolite	*K*_m_ (µM)	Scaled *V*_max_^a^ (µmol/h/g liver)	Catalytic efficiency^b^ (µL/h/g liver)	*K*_m_ (µM)	Scaled *V*_max_^a^ (µmol/h/g liver)	Catalytic efficiency^b^ (µL/h/g liver)
Conversion of estragole						
4-Allylphenol	115 ± 24	0.3	2.7	308 ± 71	1.6	5.0
Estragole-2′,3′-oxide	161 ± 17	0.9	5.5	146 ± 15	3.9	26.6
1′-Hydroxyestragole	49 ± 8	0.7	15.0	62 ± 9	2.5	40.3
3′-Hydroxyanethole	450 ± 219	0.7	1.5	429 ± 80	2.1	4.8
M5	618 ± 164	0.4	0.6	1451 ± 615	2.5	1.7
Conversion of 1′-hydroxyestragole						
1′-Hydroxyestragole glucuronide	4656 ± 1247	3.4	0.7	4607 ± 878	9.0	2.0
1′-Oxoestragole	403 ± 137	15.6	38.7	521 ± 265	24.0	46.1
1′-Sulfooxyestragole	694 ± 321	0.01	0.02	423 ± 159	0.03	0.08

^a^Scaled *V*_max_ in vivo expressed as µmol/h/(g liver), calculated from the in vitro *V*_max_ based on a microsomal protein yield of 35 mg/(g tissue) and an S9 protein yield of 143 mg/(g tissue)

^b^Catalytic efficiency expressed as µL/h/(g liver) is the ratio of scaled *V*_max_ and *K*_m_

Regarding the metabolic reactions of 1′-hydroxyestragole, formation of 1′-oxoestragole was the main detoxification pathway for this proximate carcinogenic metabolite in both populations. The catalytic efficiencies for formation of 1′-oxoestragole in both populations were similar. However, the catalytic efficiency for formation 1′-hydroxyestragole glucuronide, another detoxification route of 1′-hydroxyestragole was found to be threefold lower in incubations with Chinese than with Caucasian liver microsomes, resulting from a threefold lower *V*_max_. Sulfation of 1′-hydroxyestragole in both populations, reflecting bioactivation to a reactive ultimate carcinogen, was found to be the least efficient pathway for 1′-hydroxyestragole metabolism for both ethnic groups. For Chinese samples, the scaled catalytic efficiency for this bioactivation reaction was 4.5-fold lower than for the Caucasian samples.

### Comparison of the PBK model-based predictions for bioactivation and detoxification of estragole in Chinese and Caucasians

Comparison of the overall consequences of the observed differences in kinetic constants for all the individual bioactivation and detoxification reactions of estragole between the two ethnic groups requires integration of all data in a PBK model. In the present study, these PBK models were defined based on the PBK model for estragole in human previously developed (Punt et al. 2009), using the population specific physiological and kinetic parameters defined in the present study. The PBK model for estragole for Caucasians was evaluated previously by comparing the predicted formation of 4-allylphenol and 1′-hydroxyestragole glucuronide to the observed dose levels of these metabolites in vivo (Punt et al. 2009). The performance of the PBK model for estragole for the Chinese population could not be evaluated against Chinese in vivo data because quantitative data on the excretion of different metabolites of estragole in Chinese subjects after oral exposure to estragole are not available. Evaluation of the model could only be made by comparing the predicted inter-ethnic differences in formation of 1′-hydroxyestragole to reported ethnic differences in hepatic drug-metabolizing enzymes that mainly catalyze the formation of metabolites of estragole to 1′-hydroxyestragole. CYP1A2 and CYP2A6 predominantly catalyze formation of 1′-hydroxyestragole with CYP2C19, CYP2D6, and CYP2E1 contributing to some extent at high concentrations of estragole (Jeurissen et al. 2007). The PBK model-based predicted formation of 1′-hydroxyestragole at an oral dose of 0.01 mg/kg bw, a realistic dietary intake level (Smith et al. 2002), in Chinese liver, amounted to 43% of the dose, which was similar to the relative amount of 1′-hydroxyestragole predicted to be formed in the Caucasian liver, which was 47% of the dose as shown in Fig. 6. Although the catalytic efficiency in formation of 1′-hydroxyestragole in Chinese subjects is two to threefold lower than that of those in Caucasian incubations, the predicted overall formation of 1′-hydroxyestragole over 24 h in both ethnic groups is similar. Therefore, although the catalytic efficiency for estragole 1′-hydroxylation in the Chinese subjects is lower, when considering a time for conversion of 24 h the overall % of the dose converted to 1′-hydroxyestragole still appears to be similar. The prediction of similar overall formation of 1′-hydroxyestragole in both ethnic groups is in line with the experimental observation that peak plasma concentrations achieved at 0.5–2 h and apparent oral clearance values within 7 h of administration for phenacetin, a marker for CYP1A2 activity, did also not differ between Chinese and Caucasian subjects (Bartoli et al. 1996). At a high-dose level of 300 mg/kg bw/day estragole, the predicted formation of 1′-hydroxyestragole in the liver of Chinese subjects was twofold lower (13 versus 25% of the dose) than the predicted formation in the liver of Caucasian subjects. This would be in line with the fact that at higher estragole concentrations, CYP2C19 might become involved in estragole 1′-hydroxylation and the fact that the frequency of poor metabolizers (PM) for CYP2C19 in the Chinese populations was 14.6% compared to 3.3% in a Swedish population (Bertilsson et al. 1992). Altogether, the PBK models obtained were considered adequate for further predictions of inter-ethnic differences in estragole metabolism, also because of the previous evaluation of the model for human as reported in the literature (Punt et al. 2009).

**Fig. 5:**
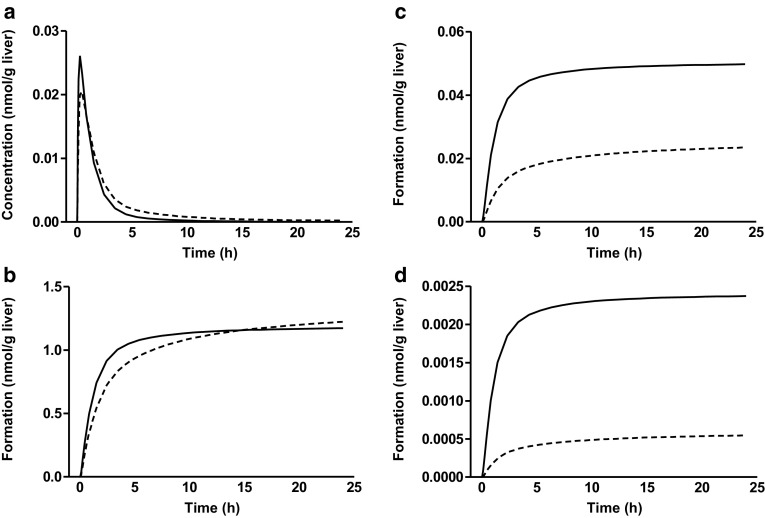
PBK model-based predictions for the time-dependent concentration of 1′-hydroxyestragole in liver (**a**), and overall formation of 1′-oxoestragole (**b**), 1′-hydroxyestragole glucuronide (**c**), and 1′-sulfooxyestragole (**d**) in Chinese (*dashed line*) and Caucasian (*continuous line*) liver following exposure to 0.01 mg/kg bw estragole

Figure 5 shows the PBK model-based predictions for the time-dependent formation of 1′-hydroxyestragole and 1′-hydroxyestragole metabolites in both ethnic groups at a dose level of 0.01 mg/kg bw/day of estragole. Although the catalytic efficiency in formation of 1′-hydroxyestragole in incubations with liver samples from Chinese subjects is two to threefold lower than that in incubations with Caucasian samples, the predicted *C*_max_ and 24 h AUC of 1′-hydroxyestragole in both the groups are similar. The predicted formation of the different metabolites amounted to 0.02 nmol/(g liver) for 1′-hydroxyestragole glucuronide, 1.2 nmol/(g liver) for 1′-oxoestragole and 0.0005 nmol/(g liver) for 1′-sulfooxyestragole in Chinese subjects, corresponding to 0.8, 42, and 0.02% of the dose, respectively (Fig. 5). For the Caucasians, these values amounted to 0.05 nmol/(g liver) for 1′-hydroxyestragole glucuronide, 1.2 nmol/(g liver) for 1′-oxoestragole and 0.002 nmol/(g liver) for 1′-sulfooxyestragole, corresponding to 2, 45 and 0.09% of the dose, respectively (Fig. 5).

**Fig. 6:**
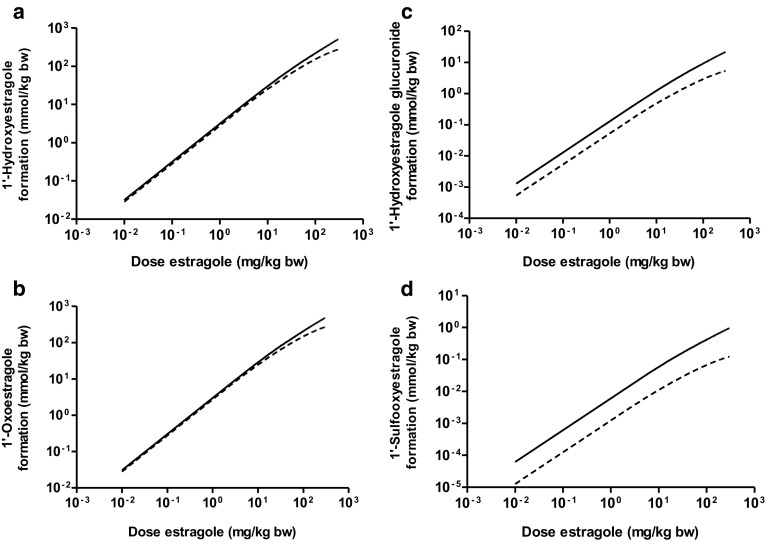
PBK model-based predictions for the dose-dependent formation of (**a**) 1′-hydroxyestragole, (**b**) 1′-oxoestragole, (**c**) 1′-hydroxyestragole glucuronide, and (**d**) 1′-sulfooxyestragole in Chinese (*dashed line*) and Caucasian (*continuous line*) liver

Figure 6 presents the PBK model-based predictions for the dose-dependent formation of 1′-hydroxyestragole and metabolites of 1′-hydroxyestragole in both ethnic groups. Differences on predicted formation of the various estragole metabolites in both ethnic groups can be considered following estragole exposure at 0.01, 5, and 150 mg/kg bw/day, representing, respectively, a realistic daily intake (Smith et al. 2002), an intake that may result from supplement use (Van Den Berg et al. 2011) and a dose level known to cause liver tumors in rodent bioassays (Drinkwater et al. 1976; Miller et al. 1983). Figure 6 shows that the ethnic differences in predicted formation of estragole metabolites in both ethnic groups at a dose of 0.01 mg/kg bw/day are similar to the situation for these metabolites formed in both ethnic groups at a dose of 5 mg/kg bw/day estragole. When the dose level increases up to 150 mg/kg bw/day, the ethnic difference in predicted metabolites of estragole increases. Figure 6a displays the predicted formation of 1′-hydroxyestragole in both ethnic groups. At dose level of 0.01 mg/kg bw/day of estragole, no ethnic difference in the formation of 1′-hydroxyestragole is observed. When the dose level increases up to 150 mg/kg bw/day, the relative formation of 1′-hydroxyestragole in both ethnic subjects decreased due to the saturation of this metabolic route. However, the formation of 1′-hydroxyestragole predicted for Chinese liver was 1.5-fold lower than for Caucasian liver. At relatively high concentrations of estragole, CYP2C19 might start to be involved in 1′-hydroxyestragole formation and a genetic polymorphism between Chinese and Caucasians has been reported with higher frequency of slow metabolizers in the Chinese population (Bertilsson et al. 1992) which may provide an explanation for the observed effect. Figure 6b presents the predicted formation of 1′-oxoestragole in both ethnic groups. Oxidation of 1′-hydroxyestragole represents the main metabolic route for 1′-hydroxyestragole in both ethnic groups at all three dose levels of estragole. The predicted formation of 1′-oxoestragole was similar at 0.01 mg/kg bw/day of estragole, 41.2% of the dose for Chinese, and 45% of the dose for Caucasians. At 5 mg/kg bw/day of estragole, 39% of dose was converted to 1′-oxoestragole in Chinese and 44% of the dose in Caucasians. When a dose of 150 mg/kg bw/day estragole was applied, only 19% of the dose was converted to 1′-oxoestragole for Chinese and 28% for Caucasians. The ethnic difference in formation of this metabolite was increased from onefold at 0.01 mg/kg bw/day to 1.5-fold at 150 mg/kg bw/day estragole. Figure 6c shows that the PBK model-based predicted formation of 1′-hydroxyestragole glucuronide in the Chinese liver was 2.4-fold lower than the levels predicted to be formed in Caucasians at a dose level of 0.01 mg/kg bw/day of estragole. At a dose level of 5 mg/kg bw/day of estragole, there was a 2.6-fold difference in formation of 1′-hydroxyestragole glucuronide between the ethnic subjects, and at a dose level of 150 mg/kg bw/day of estragole, the difference increased to 3.4-fold. Figure 6d reveals that the PBK model-based predicted formation of 1′-sulfooxestragole was 4.5-fold lower for Chinese than Caucasian liver at 0.01 mg/kg bw/day of estragole. The ethnic difference in formation of 1′-sulfooxyestragole was 5.1-fold at 5 mg/kg bw/day and 6.6-fold at 150 mg/kg bw/day of estragole pointing at lower levels of bioactivation to the ultimate carcinogenic metabolite for the Chinese population.

### Sensitivity analysis

A sensitivity analysis was performed to determine model parameters that are able to influence the formation of 1′-hydroxyestragole and 1′-sulfooxyestragole in both ethnic groups to the largest extent. Sensitivity coefficients were calculated for all model parameters at a dose of 0.01, 5, and 150 mg/kg bw/day estragole, but only the parameters that appeared to have a sensitivity coefficient higher than |0.1| are presented in Fig. S2 in supporting materials 5.

In Chinese and Caucasian liver at 0.01 mg/kg bw of estragole (Fig. S2a), formation of 1′-hydroxyestragole is predominantly influenced by the kinetic constant for 1′-hydroxylation, and to a minor extent by the kinetic constants for formation of estragole-2′,3′-oxide. Focusing on inter-ethnic differences, it can be concluded that parameters, including liver volume, blood flow to fat and microsome protein yield of liver, have higher effect on the formation of 1′-hydroxyestragole in Chinese liver compared to Caucasian liver. Formation of 1′-sulfooxyestragole mainly depends on the kinetic constants for its formation from 1′-hydroxyestragole, the formation of 1′-hydroxyestragole itself, and the kinetic constants for formation of 1′-oxoestragole, which is an important competing metabolic pathway to sulfation in both ethnic groups. At 5 mg/kg bw/day of estragole (Fig. S2b), the results of the sensitivity analysis of both ethnic groups are similar to the situation at a dose of 0.01 mg/kg bw/day. When the dose increases to 150 mg/kg bw/day (Fig. S2c), besides the influential parameters at a dose of 0.01 and 5 mg/kg bw/day of estragole, the parameters related to fat tissue, such as volume of fat and fat/blood partition coefficient, also have influence on the formation of 1′-hydroxyestragole and 1′-sulfooxyestragole in Chinese liver. For the Caucasian group, the results of the sensitivity analysis at 150 mg/kg bw/day are similar to those at 0.01 or 5 mg/kg bw/day of estragole.

## Discussion

In the present study, bioactivation and detoxification of estragole in Chinese and Caucasian populations upon oral exposure to estragole were examined using PBK modeling. The structure of the PBK models was based on the model developed and validated previously for estragole in Caucasians (Punt et al. 2009). The parameter values for the Chinese PBK model were derived from literature or from incubation experiments using human liver fractions. To guarantee adequate experimental comparison, the parameters for hepatic metabolism in the Caucasian model were redefined in the present study as well. The kinetic constants of estragole metabolism obtained from incubation with Caucasian liver fractions and PBK model predictions of estragole in Caucasians in this study were similar to results reported in the study by Punt et al. (2009).

Since the actual kinetic data on plasma or urinary estragole metabolite levels were not available for the Chinese population, the performance of the model was evaluated by comparing the predicted inter-ethnic differences in formation of 1′-hydroxyestragole to reported ethnic differences in hepatic drug-metabolizing enzymes CYP1A2 that mainly catalyze the formation of metabolites of estragole to 1′-hydroxyestragole. Furthermore, the PBK model for the Chinese population was based on the model for estragole defined and validated previously (Punt et al. 2009). In addition, the quality of the Chinese tissue samples used to determine the various kinetic parameters appeared to be in line with what has been reported before (Yang et al. 2012; Wang et al. 2006). Considering these facts, it can be concluded that the developed PBK model for the Chinese population is expected to adequately describe the in vivo levels of metabolites upon oral exposure.

The ethnic difference in bioactivation of estragole was evaluated by predicting the formation of 1′-sulfooxyestragole at doses level of 0.01 and 5 mg/kg bw/day of estragole. The PBK models predicted that following an exposure of 0.01 mg/kg bw/day estragole, only 0.02% of the estragole dose was predicted to be converted to 1′-sulfooxyestragole in the Chinese population, whereas 0.09% of the dose was converted to 1′-sulfooxyestragole in Caucasians, which was 4.5-fold higher. At 5 mg/kg bw/day of estragole, a worst case scenario intake for people using plant food supplements containing estragole (Van Den Berg et al. 2011), the difference in the predicted formation of 1′-sulfooxyestragole between the two ethnic groups was increased to fivefold. This four to fivefold difference in the level of bioactivation of estragole between the two ethnic groups mainly originates from the difference in SULT mediated conversion of 1′-hydroxyestragole. The human SULTs involved in this sulfation reaction have not been identified to date, but sulfation of the 1′-hydroxy metabolite of the related alkenylbenzene methyleugenol in the liver has been shown to be catalyzed mainly by SULT1A1 and to a small extent by SULT1E1 (Herrmann et al. 2012). It has also been shown that exon 7 of the SULT1A1 gene may contain a G to A transition at codon 213 (rs9282861), which induces an Arg to His amino acid substitution (Raftogianis et al. 1997). This SULT1A1 Arg213His (rs9282861) polymorphism is reported to be associated with increased risk of various cancer types (Xiao et al. 2014). In the ethnic subgroup analysis of Xiao et al., they found that the genotype distributions of the SNP site are different for different ethnic groups. Occurrence of the His allele in Asians (9.58%) was reported to be significantly lower than in Caucasians (35.2%). This difference of the His allele frequency could contribute to differential drug responses between these two ethnic groups. It is tempting to speculate that the SNP difference in SULT1A1 between the ethnic groups may explain the differences in formation of 1′-sulfooxyestragole as observed in the present study, although a definite conclusion should await measurement of the respective SULT reaction using samples from individuals from both genotypes.

It is of interest to consider the possible consequences of the ethnic variation in bioactivation of estragole for the related health risks. Among the different available qualitative and quantitative approaches for making such a risk assessment for a genotoxic and carcinogenic compounds, the use of the Margin of exposure (MOE) approach was advised by EFSA (EFSA 2005). The MOE is a dimensionless value that is the ratio of a reference point derived from experimental data on tumor incidence and the estimated daily intake of human. Generally, the BMDL_10_ which is the lower confidence bound of the Benchmark Dose that gives 10% extra cancer incidence above background levels can be used as the point of departure (Barlow et al. 2006). Using an estimated daily intake of 0.01 mg/kg bw/day and the BMDL_10_ of 3.3–6.5 mg/kg bw/day for estragole (Van Den Berg et al. 2011), the MOE value would amount to 330 up to 650, which is lower than 10,000, suggesting a priority for risk management. In Europe since 2008 the use of estragole as pure flavoring substance in foodstuffs has been prohibited, and for a few food categories, maximum levels of naturally occurring estragole in foodstuffs have been defined (EC 2008). When estimated daily intakes would amount to 5 mg/kg/day, for example, as a result of daily use of estragole-containing plant food supplements (Van Den Berg et al. 2011), then the MOE values would amount to 0.66 up to 1.3, indicating that the daily intake of estragole using plant food supplements is within the range of the dose levels causing tumors in the experimental animals. Considering the consequences of five-fold reduction in bioactivation of estragole at similar dose levels in the Chinese as compared to the Caucasian population, as predicted by the results of the present study, one could argue that, assuming a linear relationship between the formation of the ultimate carcinogenic metabolite and the hepatoma incidence, intake levels in the Chinese population could be fivefold higher before reaching the same risk as expected for the Caucasian population. This is confirmed by the PBK model predictions that reveal that the amount of 1′-sulfooxyestragole formed within 24 h upon an intake of, respectively, 0.01 or 5 mg/kg bw for a Caucasian would be reached upon an estragole intake of, respectively, 0.05 or 25 mg/kg bw for a Chinese individual. Converting this five-fold difference in estragole bioactivation to the MOE values presented above would still result in MOE values below 10,000 pointing at a priority for risk management also for the Chinese population. According to the national food safety standard for uses of food additives in China, estragole is allowed to be used in foods as synthetic flavoring substance and fennel oil is permitted to be used in foods as natural flavoring substance (NHFPC 2014a). The maximum levels of estragole present in foods are not well-regulated in China and thus may lead to a potential risk for the Chinese population when consuming estragole-containing food products.

It is important to note that the PBK model used in the present study was developed to predict the formation of the ultimate carcinogen metabolite, 1′-sulfooxyestragole. Further metabolic reactions of 1′-sulfooxyestragole such as conjugation with GSH, RNA, DNA, and other cellular macromolecules were not included in the PBK model. Possible ethnic differences in the capacity of DNA repair and reversibility of DNA-adduct formation may also be present. For example, genetic polymorphisms in DNA repair genes ERCC1 and ERCC2/XPD, which are involved in the nucleotide excision repair pathway, have been identified (Yin et al. 2005). Previous studies reported that the A-allele frequency of ERCC1 G19007A in the northeastern Chinese population was much lower compared with Caucasian, indicating another genetic contribution to differences in sensitivity to genotoxic carcinogens between ethnic groups (Yin et al. 2005). The current PBK model could be extended to a PBD model by including DNA-adduct formation in hepatocytes from Chinese donors, which allows taking into account possible ethnic difference in DNA repair.

In summary, the present study elucidates a possible approach to determine the inter-ethnic human difference in bioactivation and detoxification of compounds of concern taking estragole as the model compound. The outcomes obtained clearly illustrate that PBK modeling is essential to integrate information on ethnic differences in the various metabolic conversions to enable prediction of their combined consequence for the bioactivation to the ultimate carcinogenic metabolite. The 4.5-fold difference in formation of the ultimate carcinogenic metabolite of estragole accompanied by similar rates of detoxification may indicate a lower risk of estragole for the Chinese population at similar levels of exposure. The study provides a proof of principle for how PBK modeling can identify ethnic sensitivity and provide a more refined risk assessment for a specific ethnic group for a compound of concern.

## Electronic supplementary material

Below is the link to the electronic supplementary material.

Supplementary material 1 (DOCX 23 KB)

Supplementary material 2 (DOCX 30 KB)

Supplementary material 3 (DOCX 20 KB)

Supplementary material 4 (DOCX 90 KB)

Supplementary material 5 (DOCX 172 KB)

Supplementary material 6 (DOCX 25 KB)
