# Supplementary material for: Study on inter-ethnic human differences in bioactivation and detoxification of estragole using physiologically based kinetic modeling
Source: Arch Toxicol. 2017 Mar 29;91(9):3093–108. doi: 10.1007/s00204-017-1941-x (PMC5562778; doi:10.1007/s00204-017-1941-x)
Supplement: Supplementary file 1 — Supplementary material 1 (DOCX 23 KB) [file 204_2017_1941_MOESM1_ESM.docx]

**Study on inter-ethnic human differences in bioactivation and detoxification of estragole using physiologically based kinetic modelling**

Jia Ning ^*1^, Jochem Louisse ^1^, Bert Spenkelink ^1^, Sebastiaan Wesseling^1^, Ivonne M.C.M. Rietjens^1^

**^1)^** Division of Toxicology, Wageningen University, Stippeneng 4, 6708 WE Wageningen, The Netherlands

^*^Corresponding author:

Jia Ning

Division of Toxicology, Wageningen University

Stippeneng 4, 6708 WE Wageningen, the Netherlands

Tel: +31-317 484357

Fax: +31-317 484931

Email: jia.ning@wur.nl

**Supporting materials 1**

**Material and methods**

**Assessment of metabolic capabilities of CYP and SULT enzymes in Chinese and Caucasian liver samples**

***In vitro incubations***

The quality of the Chinese and Caucasian liver microsomes was checked by measuring the activity of CYP 1A2 and CYP 2A6 by the method of Yang et al. (Yang et al. 2012). CYP 1A2 and CYP 2A6 are predominant enzymes for formation of 1'-hydroxyestragole (Jeurissen et al. 2007). The activity of CYP 1A2 was measured with the substrate phenacetin, and the activity of CYP 2A6 was checked using the substrate coumarin. Briefly, the incubations were performed in triplicate containing 0.1 mg/ml microsomal protein in 0.1 M potassium phosphate pH 7.4 (for CYP 1A2) / 0.1 M Tris buffer pH 7.4 (for CYP 2A6) with (final concentrations) 10 to 2000 μM phenacetin or 1.6 to 50 μM coumarin added from 100 times concentrated stock solutions in DMSO in the presence of an NADPH-generating system consisting of (final concentrations) 3.3 mM of glucose-6-phosphate, 0.5 U/ml glucose-6-phosphate dehydrogenase and 1.3 mM NADP^+^. Before adding the NADPH-generating system, the mixture was incubated for 3 min. The optimal incubation time was 20 min for phenacetin *O*-deethylation and coumarin 7-hydroxylation (Yang et al. 2012). The reactions were terminated by adding 100 μl of ice-cold methanol. The samples were centrifuged for 5 min at 16, 000g to precipitate microsomal proteins and analyzed by UPLC.

The quality of Chinese and Caucasian liver S9 samples was checked by measuring the SULT activity based on the method provided by Wang et al. (Wang et al. 2006). Incubations with Chinese liver S9 were performed in 100 μl incubation mixture containing (final concentrations) 1 mM PAPS, 0.4 mg/ml S9 and the substrate 7-hydroxycoumarin at concentrations ranging from 1.5 to 25 μM in 0.1 M Tris buffer (pH 7.4). Incubations were carried out for 10 min and the reactions were terminated by adding 25 μl ice-cold acetonitrile. The samples were centrifuged for 5 min at 16, 000 g to precipitate proteins and were analyzed by UPLC.

**UPLC analysis of conversion of phenacetin, coumarin and 7-hydroxycoumarin in incubations with Chinese and Caucasian liver samples**

Before UPLC analysis, all samples were centrifuged for 5 min at 16, 000g to precipitate microsomal or cytosolic proteins. Supernatant of each sample was analyzed on UPLC using a BEH C18 (1.7 μm 2.1×50 mm) column with a guard column and a diode array detector (Acquity, Waters). The flow rate was 0.6 ml/min and the mobile phase was made with ultrapure water with 0.1 % (v/v) TFA and acetonitrile. Identification of metabolites of phenacetin, coumarin and 7-hydroxycoumarin was achieved by comparison of the UV spectra and retention times of formed metabolites to those of commercially available reference compounds. Quantification of metabolites was done by comparing the peak areas of formed metabolites to the calibration curve of the corresponding standard compounds. For the analysis of acetaminophen formed by O-deethylation of phenacetin to check the activity of CYP 1A2, a gradient was applied from 5 to 30 % acetonitrile in ultrapure water in 5 min, after which the percentage of acetonitrile was increased to 70 % in 1 min and kept at this level for 1 min longer. Under these conditions, acetaminophen had a retention time of 0.7 min. For detection of 7-hydroxycoumarin by hydroxylation of coumarin to check the activity of CYP 2A6, the gradient started with 20 % acetonitrile in ultrapure water for 0.3 min, followed by a linear gradient from 20 to 30 % during 0.7 min, and a subsequent increase to 80 % acetonitrile in 1.5 min. This percentage was kept for 0.5 min, after which the column was set back to the starting conditions. Under these conditions 7-hydroxycoumarin eluted at 0.6 min. For measuring the sulfate metabolite of 7-hydroxycoumarin to check SULT activity in Chinese and Caucasian liver S9, the gradient was started from 20 to 30 % acetonitrile in 4.5 min, after which the percentage of acetonitrile was increased to 100 % in 0.3 min and kept at 100 % for another 0.2 min. Under these conditions, 7-hydroxycoumarin sulfate had a retention time of 1 min.

**Reference**

Jeurissen SM, Punt A, Boersma MG, Bogaards JJ, Fiamegos YC, Schilter B, van Bladeren PJ, Cnubben NH, Rietjens IM (2007) Human cytochrome P450 enzyme specificity for the bioactivation of estragole and related alkenylbenzenes. Chem Res Toxicol 20(5):798-806

Wang Q, Ye C, Jia R, Owen AJ, Hidalgo IJ, Li J (2006) Inter-species comparison of 7-hydroxycoumarin glucuronidation and sulfation in liver S9 fractions. In Vitro Cell Dev Biol Anim 42(1-2):8-12

Yang J, He MM, Niu W, Wrighton SA, Li L, Liu Y, Li C (2012) Metabolic capabilities of cytochrome P450 enzymes in Chinese liver microsomes compared with those in Caucasian liver microsomes. Brit J Clin Pharmaco 73(2):268-284
