# Supplementary material for: Study on inter-ethnic human differences in bioactivation and detoxification of estragole using physiologically based kinetic modeling
Source: Arch Toxicol. 2017 Mar 29;91(9):3093–108. doi: 10.1007/s00204-017-1941-x (PMC5562778; doi:10.1007/s00204-017-1941-x)
Supplement: Supplementary file 2 — Supplementary material 2 (DOCX 30 KB) [file 204_2017_1941_MOESM2_ESM.docx]

**Study on inter-ethnic human differences in bioactivation and detoxification of estragole using physiologically based kinetic modelling**

Jia Ning ^*1^, Jochem Louisse ^1^, Bert Spenkelink ^1^, Sebastiaan Wesseling^1^, Ivonne M.C.M. Rietjens^1^

**^1)^** Division of Toxicology, Wageningen University, Stippeneng 4, 6708 WE Wageningen, The Netherlands

^*^Corresponding author:

Jia Ning

Division of Toxicology, Wageningen University

Stippeneng 4, 6708 WE Wageningen, the Netherlands

Tel: +31-317 484357

Fax: +31-317 484931

Email: jia.ning@wur.nl

**Supporting materials 2**

**Material and methods**

Table S1 tracks the detailed methods for in vitro incubations with estragole and 1'-hydroxyestragole and for UPLC analysis of metabolite formation for obtaining the kinetic parameters.

**Table S1** The kinetic parameters of metabolites of estragole and 1'-hydroxyestragole were derived from corresponding methods of in vitro incubations and UPLC analysis

| Metabolites | Kinetic parameters | In vitro incubations | UPLC analysis |
| --- | --- | --- | --- |
| 4-allylphenol  1'-hydroxyestragole  estragole-2',3'-oxide  3'-hydroxyanethole  M5 | V_max_, _L_AP_, K_max_, _L_AP_  V_max_, _L_HE_, K_max_, _L_HE_  V_max_, _L_EE_, K_max_, _L_EE_  V_max_, _L_HA_, K_max_, _L_HA_  V_max_, _L_M5_, K_max_, _L_M5_ | Microsomal metabolism of estragole | UPLC analysis of estragole metabolites |
| 1'-hydroxyestragole  glucuronide | V_max_, _L_HEG_, K_max, L_HEG_ | Glucuronidation of 1'-hydroxyestragole | UPLC analysis of 1'-hydroxyestragole metabolites |
| 1'-oxoestragole | V_max_, _L_OE_, K_max_, _L_OE_ | Oxidation of 1'-hydroxyestragole | UPLC analysis of 1'-hydroxyestragole metabolites |
| 1'-sulfooxyestragole | V_max_, _L_HES_, K_max_, _L_HES_ | Sulfation of 1'-hydroxyestragole | UPLC analysis of 1'-hydroxyestragole metabolites |

**In vitro incubations**

***Microsomal metabolism of estragole***

The kinetic constants for the microsomal conversion of estragole were determined as previously described by Punt et al. (Punt et al. 2009). Briefly, mixed gender Chinese or Caucasian liver microsomes were incubated with estragole in the presence of NADPH. The incubation mixtures contained (final concentrations) 3 mM NADPH and 1 mg/ml microsomal protein in 0.2 M Tris-HCl (pH 7.4). After 1 min pre-incubation at 37 °C, the reaction was started by addition of the substrate estragole from 100 times concentrated stock solutions in DMSO. Incubations were performed for 10 min at substrate concentrations ranging from 25 to 1000 μM, after which the reaction was terminated by adding 25 μl ice-cold acetonitrile. Under these conditions, the formation of the different estragole metabolites was linear with time and microsomal protein concentration (Punt et al. 2009). Blank incubations were performed in the absence of the cofactor NADPH. All incubations were performed in triplicate.

***Glucuronidation of 1'-hydroxyestragole***

Pooled mixed gender Chinese or Caucasian liver microsomes were incubated with 1'-hydroxyestragole in the presence of UDPGA. As previously described by Punt et al. (Punt et al. 2009), the incubation mixtures contained (final concentrations) 10 mM UDPGA, and 1 mg/ml microsomal protein in 0.2 M Tris-HCl (pH 7.4) with 10 mM MgCl_2_. To overcome enzyme latency and obtain maximal glucuronidation activity (Fisher et al. 2000; Lin and Wong 2002), the mixtures were pre-treated on ice for 15 min with 0.025 mg/ml of the pore forming peptide alamethicin added from a 200 times concentrated stock in methanol. After 1 min pre-incubation at 37 °C, the reactions were started by adding the substrate 1'-hydroxyestragole from 100 times concentrated stock solutions in DMSO at final concentrations ranging from 60-2000 μM. Incubations were carried out for 6 hours and the reaction was terminated by adding 25 μl ice-cold acetonitrile. Under these conditions, the formation of the glucuronide of 1'-hydroxyestragole was linear with time and microsomal protein concentration (Punt et al. 2009). Blank incubations were performed in the absence of the cofactor UDPGA. All incubations were performed in triplicate.

***Oxidation of 1'-hydroxyestragole***

Mixed gender Chinese or Caucasian liver S9 was incubated with 1'-hydroxyestragole in the presence of NAD^+^ and GSH, the latter added to trap the transient 1'-oxoestragole. Formation of the 1'-oxoestragole adducts with GSH forming GS-1'-oxoestragole reflects the formation of 1'-oxoestragole (Punt et al. 2009). The incubations had a final volume of 100 μl, containing (final concentrations) 3 mM NAD^+^, 2 mM GSH and 1 mg/ml liver S9 in 0.2 M Tris-HCl (pH 7.4), as described previously by Punt et al. (Punt et al. 2016). The mixtures were pre-incubated for 1 min at 37 °C, after which the reactions were started by adding the substrate 1'-hydroxyestragole at concentrations ranging from 15 to 1000 μM added from 100 times concentrated stock solutions in DMSO. The reactions were terminated after 10 min by the addition of 25 μl ice-cold acetonitrile. Under these conditions, the formation of the GSH adduct of 1'-oxoestragole was linear with time and cytosolic protein concentration (Punt et al. 2009). Blank incubations were performed without cofactor NAD^+^. All incubations were performed in triplicate.

***Sulfation of 1'-hydroxyestragole***

The formation of 1'-sulfooxyestragole was determined by incubating 0.2 mg/ml pooled mixed gender Chinese or Caucasian liver S9 in the presence of 0.2 mM PAPS as cofactor and 10 mM GSH as trapping agent for the reactive 1'-sulfooxyestragole in 0.1 M potassium phosphate (pH 8.0). The final volume of the incubations was 100 μl with the substrate 1'-hydroxyestragole ranging from 15 to 2000 μM added from 100 times concentrated stock solutions in DMSO. As previously described by Martati et al. (Martati et al. 2011) and Al-Subeihi et al. (Al-Subeihi et al. 2011), the reactions were started by adding substrate after a pre-incubation at 37 °C for 1 min. The incubations were carried out for 2 hours and the reactions were terminated by adding 25 μl ice-cold acetonitrile. Under these conditions, the formation of the GSH adduct of 1'-sulfooxyestragole was linear with time and microsomal protein concentration (Martati et al. 2011). The blank samples were performed without cofactor. All incubations were performed in triplicate.

**UPLC analysis**

***UPLC analysis of estragole metabolites***

For detection of microsomal estragole metabolites by UPLC, the gradient was made with 100 % ultrapure water and 100 % acetonitrile. The flow rate was 0.6 ml/min and the gradient started with 100 % ultrapure water. After 1 min, the amount of acetonitrile was increased to 10 % and kept at that level for 1 min, after which the percentage of acetonitrile was increased to 35 % in 2.5 min and then increased to 100 % over 0.5 minute and kept at 100 % acetonitrile for another 1 min, and finally back to the starting conditions.

***UPLC analysis of 1'-hydroxyestragole metabolites***

The gradient for analysis of 1'-hydroxyestragole glucuronide was performed with ultrapure water containing 0.1 % (v/v) TFA and 100 % acetonitrile with a 0.6 ml/min flow rate. A gradient was applied from 10 to 25 % acetonitrile over 3.5 min, after which the acetonitrile was increased to 100 % in 0.5 min, kept at 100 % for 0.5 min and finally set back to the starting condition.

The gradient for analysis of the metabolites contains a mixture of acetonitrile and 0.1 % TFA in ultrapure water with 0.6 ml/min flow rate. A gradient from 0 to 25 % acetonitrile was applied over 1.2 min and kept at 25 % acetonitrile for 0.2 min, after which the percentage of acetonitrile was increased to 100 % in 0.5 min and then kept at 100 % for another 0.6 min.

For detection of the GSH conjugate of 1'-sulfooxyestragole, the flow rate was 0.6 ml/min and the mobile phase was made with acetonitrile and ultrapure water with 0.1 % (v/v) TFA. A linear gradient started from 20 to 30 % acetonitrile in 4.5 min, after which the percentage of acetonitrile was increased to 100 % in 0.3 min and kept at 100 % for another 0.2 min and finally set back to the starting condition.

**Reference**

Al-Subeihi AA, Spenkelink B, Rachmawati N, Boersma MG, Punt A, Vervoort J, van Bladeren PJ, Rietjens IM (2011) Physiologically based biokinetic model of bioactivation and detoxification of the alkenylbenzene methyleugenol in rat. Toxicol in Vitro 25(1):267-285

Fisher MB, Campanale K, Ackermann BL, VandenBranden M, Wrighton SA (2000) In vitro glucuronidation using human liver microsomes and the pore-forming peptide alamethicin. Drug Metab Dispos 28(5):560-566

Lin JH, Wong BK (2002) Complexities of glucuronidation affecting in vitro-in vivo extrapolation. Curr Drug Metab 3(6):623-646

Martati E, Boersma MG, Spenkelink A, Khadka DB, Punt A, Vervoort J, van Bladeren PJ, Rietjens IMCM (2011) Physiologically based biokinetic (PBBK) model for safrole bioactivation and detoxification in rats. Chem Res Toxicol 24(6):818-834

Punt A, Paini A, Boersma MG, Freidig AP, Delatour T, Scholz G, Schilter B, van Bladeren PJ, Rietjens I (2009) Use of Physiologically Based Biokinetic (PBBK) Modeling to Study Estragole Bioactivation and Detoxification in Humans as Compared with Male Rats. Toxicol Sci 110(2):255-269

Punt A, Paini A, Spenkelink A, Scholz G, Schilter B, van Bladeren P, Rietjens IMCM (2016) Evaluation of interindividual human variation in bioactivation and DNA adduct formation of estragole in liver predicted by physiologically based kinetic/dynamic (PBK/D) and Monte Carlo modeling. Chem Res Toxicol 29(4):659–668
