# Supplementary material for: Study on inter-ethnic human differences in bioactivation and detoxification of estragole using physiologically based kinetic modeling
Source: Arch Toxicol. 2017 Mar 29;91(9):3093–108. doi: 10.1007/s00204-017-1941-x (PMC5562778; doi:10.1007/s00204-017-1941-x)
Supplement: Supplementary file 3 — Supplementary material 3 (DOCX 20 KB) [file 204_2017_1941_MOESM3_ESM.docx]

**Study on inter-ethnic human differences in bioactivation and detoxification of estragole using physiologically based kinetic modelling**

Jia Ning ^*1^, Jochem Louisse ^1^, Bert Spenkelink ^1^, Sebastiaan Wesseling^1^, Ivonne M.C.M. Rietjens^1^

**^1)^** Division of Toxicology, Wageningen University, Stippeneng 4, 6708 WE Wageningen, The Netherlands

^*^Corresponding author:

Jia Ning

Division of Toxicology, Wageningen University

Stippeneng 4, 6708 WE Wageningen, the Netherlands

Tel: +31-317 484357

Fax: +31-317 484931

Email: jia.ning@wur.nl

**Supporting materials 3**

**Results**

**Metabolic capabilities of CYP and SULTs enzymes**

The quality of the Chinese and Caucasian liver microsomes was checked by measuring CYP 1A2 mediated O-deethylation of phenacetin, and CYP 2A6 mediated 7-hydroxylation of coumarin. The catalytic efficiency (V_max_/K_m_) for CYP 1A2 mediated O-deethylation of phenacetin, and CYP 2A6 mediated 7-hydroxylation of coumarin by Chinese liver microsomes were 3.1 and 525.8 μl/min/(mg microsomal protein), respectively, which were in the range of results reported before in the literature showing a catalytic efficiency of 0.6-51.4 μl/min/(mg microsomal protein) for CYP 1A2 and of 150-1776 μl/min/(mg microsomal protein) for CYP 2D6 in Chinese liver microsomes (Yang et al. 2012). These catalytic efficiencies for the CYP 1A2 and CYP 2D6 mediated conversions by Chinese liver microsomes were 3- and 1.6-fold lower than the corresponding catalytic efficiency values by Caucasian liver microsomes, which was consistent with the data reported by Yang et al. (Yang et al. 2012) indicating a 2-fold lower value for catalytic efficiency of CYP 1A2 for Chinese liver microsomes and no difference between Chinese and Caucasian liver microsomes in the case of CYP 2A6 catalyzed activity.

The quality of the Chinese and Caucasian liver S9 preparations was checked by measuring the kinetics for SULT mediated conversion of 7-hydroxycoumarin to 7-hydroxycoumarin sulfate. The K_m_ for SULT mediated conversion of 7-hydroxycoumarin in incubations with Chinese liver S9 was 1.7 μM and thus similar to reported data by Wang et al. (Wang et al. 2006) and the V_max_ for both ethic groups appeared to be similar which was also in line with previously reported data (Wang et al. 2006).

**Reference**

Wang Q, Ye C, Jia R, Owen AJ, Hidalgo IJ, Li J (2006) Inter-species comparison of 7-hydroxycoumarin glucuronidation and sulfation in liver S9 fractions. In Vitro Cell Dev Biol Anim 42(1-2):8-12

Yang J, He MM, Niu W, Wrighton SA, Li L, Liu Y, Li C (2012) Metabolic capabilities of cytochrome P450 enzymes in Chinese liver microsomes compared with those in Caucasian liver microsomes. Brit J Clin Pharmaco 73(2):268-284
