# Supplementary material for: Study on inter-ethnic human differences in bioactivation and detoxification of estragole using physiologically based kinetic modeling
Source: Arch Toxicol. 2017 Mar 29;91(9):3093–108. doi: 10.1007/s00204-017-1941-x (PMC5562778; doi:10.1007/s00204-017-1941-x)
Supplement: Supplementary file 4 — Supplementary material 4 (DOCX 90 KB) [file 204_2017_1941_MOESM4_ESM.docx]

**Study on inter-ethnic human differences in bioactivation and detoxification of estragole using physiologically based kinetic modelling**

Jia Ning ^*1^, Jochem Louisse ^1^, Bert Spenkelink ^1^, Sebastiaan Wesseling^1^, Ivonne M.C.M. Rietjens^1^

**^1)^** Division of Toxicology, Wageningen University, Stippeneng 4, 6708 WE Wageningen, The Netherlands

^*^Corresponding author:

Jia Ning

Division of Toxicology, Wageningen University

Stippeneng 4, 6708 WE Wageningen, the Netherlands

Tel: +31-317 484357

Fax: +31-317 484931

Email: jia.ning@wur.nl

**Supporting materials 4**

**Results**


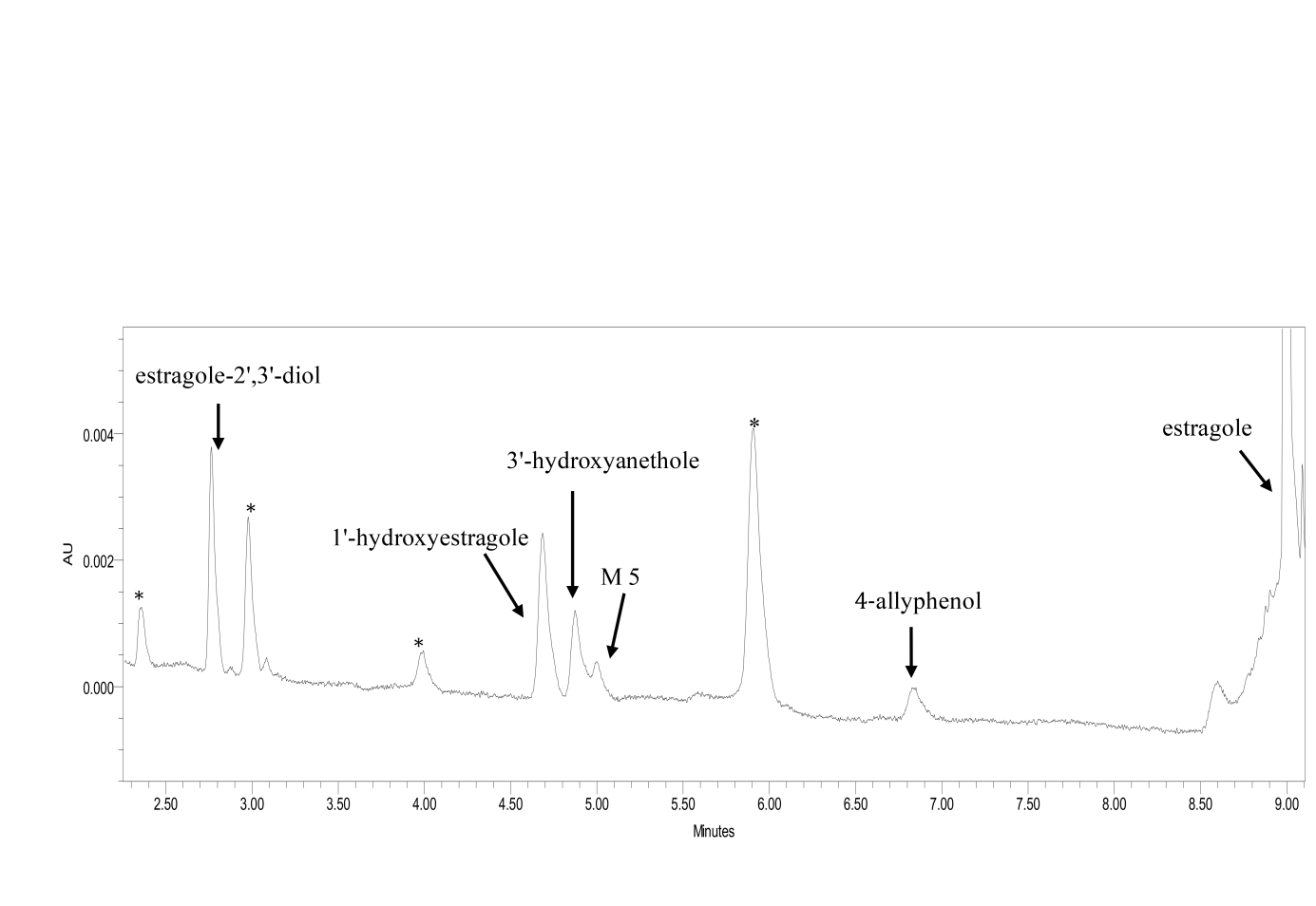


**Fig. S1** UPLC chromatogram of an incubation of estragole with Chinese liver microsomes and NADPH. Peaks marked with an asterisk were also present in the blank incubations without NADPH
