# Supplementary material for: Study on inter-ethnic human differences in bioactivation and detoxification of estragole using physiologically based kinetic modeling
Source: Arch Toxicol. 2017 Mar 29;91(9):3093–108. doi: 10.1007/s00204-017-1941-x (PMC5562778; doi:10.1007/s00204-017-1941-x)
Supplement: Supplementary file 5 — Supplementary material 5 (DOCX 172 KB) [file 204_2017_1941_MOESM5_ESM.docx]

**Study on inter-ethnic human differences in bioactivation and detoxification of estragole using physiologically based kinetic modelling**

Jia Ning ^*1^, Jochem Louisse ^1^, Bert Spenkelink ^1^, Sebastiaan Wesseling^1^, Ivonne M.C.M. Rietjens^1^

**^1)^** Division of Toxicology, Wageningen University, Stippeneng 4, 6708 WE Wageningen, The Netherlands

^*^Corresponding author:

Jia Ning

Division of Toxicology, Wageningen University

Stippeneng 4, 6708 WE Wageningen, the Netherlands

Tel: +31-317 484357

Fax: +31-317 484931

Email: jia.ning@wur.nl

**Supporting materials 5**

**Results**

Fig. S2 Sensitivity analysis for the predicted formation of 1'-hydroxyestragole and 1'-sulfooxyestragole in the liver using the Chinese (black bar) and Caucasian (white bar) PBK model at a dose of 0.01 mg/kg bw (a), 5 mg/kg bw (b) and 150 mg/kg bw (c) of estragole. The list of parameters is as follows: liver volume (VL), blood flow to the fat (QF), scaling factor of liver microsomal protein (MPL), the maximum rate and the Michaelis-Menten constant for formation of 1'-hydroxyestragole (V_max_, L_HE and K_m_, L_HE), estragole-2',3'-oxide (V_max_, L_EE and K_m_, L_EE), 1'-oxoestragole (V_max_, L_OE and K_m_, L_OE) and 1'-sulfooxyestragole (V_max_, L_HES and K_m_, L_HES) in the liver. Only the parameters for which at least one of the model appeared to have a sensitivity coefficient higher than |0.1| are presented
