# Supplementary material for: Study on inter-ethnic human differences in bioactivation and detoxification of estragole using physiologically based kinetic modeling
Source: Arch Toxicol. 2017 Mar 29;91(9):3093–108. doi: 10.1007/s00204-017-1941-x (PMC5562778; doi:10.1007/s00204-017-1941-x)
Supplement: Supplementary file 6 — Supplementary material 6 (DOCX 25 KB) [file 204_2017_1941_MOESM6_ESM.docx]

**Study on inter-ethnic human differences in bioactivation and detoxification of estragole using physiologically based kinetic modelling**

Jia Ning ^*1^, Jochem Louisse ^1^, Bert Spenkelink ^1^, Sebastiaan Wesseling^1^, Ivonne M.C.M. Rietjens^1^

**^1)^** Division of Toxicology, Wageningen University, Stippeneng 4, 6708 WE Wageningen, The Netherlands

^*^Corresponding author:

Jia Ning

Division of Toxicology, Wageningen University

Stippeneng 4, 6708 WE Wageningen, the Netherlands

Tel: +31-317 484357

Fax: +31-317 484931

Email: jia.ning@wur.nl

**Supporting materials 6**

{Berkeley Madonna code PBK model estragole Chinese}

;================================================================================

;Physiological parameters

;================================================================================

;Tissue volumes

BW = 60 {Kg} ; body weight human

VLc = 0.023 ; fraction of liver tissue

VFc = 0.187 ; fraction of fat tissue

VAc = 0.02 ; fraction of arterial blood: 0.079*1/4

VVc = 0.059 ; fraction of venous blood: 0.079*3/4

VRc = 0.076-VLc ; fraction of richly perfused tissue

VSc = 0.81-VFc-VAc-VVc ; Fraction of blood flow to slowly perfused tissue

; total of fractions = 0.886

VL = VLc*BW {L or Kg}

VF = VFc*BW

VR = VRc*BW

VS = VSc*BW

VA = VAc*BW

VV = VVc*BW

;-----------------------------------------------------------------------------------------------------------------------------------------

;Blood flow rates

QC = 15*BW**0.74 {L/hr} ; Info: QC=15*BW^0.74

QLc = 0.2625 ; Fraction of blood flow to liver

QFc = 0.0675 ; Fraction of blood flow to fat

QRc = 0.70-QL_C_ ; Fraction of blood flow to richly perfused tissue

QSc = 0.30-QF_C_ ; Fraction of blood flow to slowly perfused tissue

; total of fractions = 1

QL = QLc*QC {L/hr}

QK = QKc*QC

QF = QFc*QC

QR = QRc*QC

QS = QSc*QC

;================================================================================

;Partition Coefficients

;================================================================================

;estragole

PLE = 6.5 ; liver/blood partition coefficient

PFE = 105 ; fat/blood partition coefficient

PRE = 6.5 ; richly perfused tissues/blood partition coefficient

PSE = 4.1 ; slowly perfused tissues/blood partition coefficient

;1'-hydroxyestragole

PLHE = 1.6 ;liver/blood partition coefficient

;================================================================================

;Biochemical parameters

;================================================================================

;Linear uptake rate (hr-1)

Ka = 1

;-----------------------------------------------------------------------------------------------------------------------------------------

;Metabolism liver

;Scaling factors

S9PL=143; Liver S9 protein yield (mg/gram liver)

MPL=35; Liver microsomal protein yield (mg/gram liver)

L=VLC*1000; Liver = 23 (gram/kg BW)

;metabolites of estragole, unscaled maximum rate of metabolism (nmol min-1 (mg protein)-1)

VmaxLHEc = 0.35 ;HE = 1'-hydroxyestragole,

VmaxLAPc = 0.15 ;AP = 4-allylphenol

VmaxLEEc = 0.42 ;EE = estragole-2',3'-oxide

VmaxLHAc = 0.32 ;HA = 3'-hydroxyanethole

VmaxLM5c = 0.18 ;M5 = metabolites 5

;metabolites of estragole, scaled maximum rate of metabolism (umol hr-1)

VMaxLHE = VMaxLHEc/1000*60*MPL*L*BW

VMaxLAP = VMaxLAPc/1000*60*MPL*L*BW

VMaxLEE = VMaxLEEc/1000*60*MPL*L*BW

VMaxLHA = VMaxLHAc/1000*60*MPL*L*BW

VMaxLM5 = VMaxLM5c/1000*60*MPL*L*BW

;metabolites of estragole, affinity constants (umol/L)

KmLHE = 49

KmLAP = 115

KmLEE = 161

KmLHA = 450

KmLM5 = 618

;metabolites of 1'-hydroxyestragole, unscaled maximum rate of metabolism (nmol min-1 (mg protein)-1)

VmaxLHEGc = 1.63 ; HEG = 1'-hydroxyestragole glucuronide

VmaxLOEc = 1.82 ; OE= 1'-oxoestragole

VmaxLHESc = 0.0014 ; HES = 1'-sulfooxyestragole

;metabolites of 1'-hydroxyestragole, scaled maximum rate of metabolism (umol hr-1)

VMaxLHEG = VmaxLHEGc/1000*60*MPL*L*BW

VMaxLOE = VmaxLOEc/1000*60*S9PL*L*BW

VMaxLHES = VmaxLHESc/1000*60*S9PL*L*BW

;metabolites of 1'-hydroxyestragole, affinity constants (umol/L)

KmLHEG = 4656

KmLOE = 403

KmLHES = 694

;================================================================================

;Run settings

;================================================================================

;Molecular weight

MWE = 148.2; Molecular weight estragole

MWHE=164.2; Molecular weight 1'-hydroxyestragole

;Given dose (mg/kg bw) and oral dose umol/kg bw}

GDOSE = 0.01 {mg/kg bw} ; GDOSE = given dose

ODOSE = (GDOSE*1E-3)/MWE*1E6 {umol/kg bw} ; ODOSE = given dose recalculated to umol/kg bw

DOSE=ODOSE*BW; ; DOSE = umol

;Time

Starttime = 0; in hrs

Stoptime = 24; in hrs

;================================================================================

;Dynamics

;================================================================================

;slowly perfused tissue compartment

;AS = Amount estragole in slowly perfused tissue, umol

AS' = QS*(CA-CVS)

Init AS = 0

CS = AS/VS

CVS = CS/PSE

;-----------------------------------------------------------------------------------------------------------------------------------------

;richly perfused tissue compartment

;AR = Amount estragole in richly perfused tissue, umol

AR' = QR*(CA-CVR)

Init AR = 0

CR = AR/VR

CVR = CR/PRE

;-----------------------------------------------------------------------------------------------------------------------------------------

;fat compartment

;AF = Amount estragole in fat tissue, umol

AF' = QF*(CA-CVF)

Init AF = 0

CF = AF/VF

CVF = CF/PFE

;-----------------------------------------------------------------------------------------------------------------------------------------

;uptake estragole from GI tract

;AGI = Amount estragole remaining in GI tract (umol)

AGI' =-Ka*AGI

Init AGI = DOSE

;-----------------------------------------------------------------------------------------------------------------------------------------

;liver compartment

;estragole

;AL = Amount Estragole in liver tissue, umol

AL' = QL*(CA -CVL)+ Ka*AGI - AMLHE' - AMLAP' - AMLEE' -AMLHA'-AMLM5'

Init AL = 0

CL = AL/VL

CVL = CL/PLE

AUCL' = CL

Init AUCL = 0

;AMLHE=Amount estragole metabolized in liver to 1'-hydroxyestragole (HE)

AMLHE' = VmaxLHE*CVL/(KmLHE + CVL)

Init AMLHE = 0

CMLHE =AMLHE/VL

;AMLAP=Amount estragole metabolized in liver to 4-allylphenol (AP)

AMLAP' = VmaxLAP*CVL/(KmLAP + CVL)

Init AMLAP = 0

CMLAP =AMLAP/VL

;AMLEE=Amount estragole metabolized in liver to estragole-2',3'-oxide (EE)

AMLEE' = VmaxLEE*CVL/(KmLEE + CVL)

Init AMLEE = 0

CMLEE =AMLEE/VL

;AMLHA=Amount estragole metabolized in liver to 3'-hydroxyanethole (HA)

AMLHA' = VmaxLHA*CVL/(KmLHA + CVL)

Init AMLHA = 0

CMLHA =AMLHA/VL

;AMLM5=Amount estragole metabolized in liver to M5 (M5)

AMLM5' = VmaxLM5*CVL/(KmLM5 + CVL)

Init AMLM5 = 0

CMLM5 =AMLM5/VL

;1'-hydroxyestragole

;ALHE = amount 1'-hydroxyestragole in liver tissue, umol

ALHE' = AMLHE' - AMLHEG' - AMLHES' - AMLOE'

Init ALHE = 0

CLHE = ALHE/VL

CVLHE = CLHE/PLHE

;AMLHEG= amount 1'-hydroxyestragole metabolized in liver to 1'-hydroxyestragole glucurondie (HEG)

AMLHEG' = VmaxLHEG*CVLHE/(KmLHEG + CVLHE)

Init AMLHEG = 0

CLHEG = AMLHEG/VL

;AMLOE= amount 1'-hydroxyestragole metabolized in liver to 1'-oxoestragole (OE)

AMLOE' = VmaxLOE*CVLHE/(KmLOE + CVLHE)

Init AMLOE = 0

CLOE = AMLOE/VL

;AMLHES= amount 1'-hydroxyestragole metabolized in liver to 1'-sulfooxyestragole (HES)

AMLHES' = VmaxLHES*CVLHE/(KmLHES + CVLHE)

Init AMLHES = 0

CLHES = AMLHES/VL

;-----------------------------------------------------------------------------------------------------------------------------------------

; arterial blood compartment

;CA = Concentration arterial blood estragole

CA=CV

;-----------------------------------------------------------------------------------------------------------------------------------------; venous blood compartment

;CV = Concentration venous blood estragole (umol/L)

AV' = (QF*CVF + QR*CVR + QS*CVS + QL*CVL - QC*CV)

Init AV = 0

CV = AV/VV

AUCV' = CV

Init AUCV = 0

;================================================================================

;Mass balance calculations

;================================================================================

{Mass Balance}

Total = DOSE

Calculated = AF + AS + AR + AL + AV+ AGI + AMLEE + AMLHA+ AMLHE + AMLAP+ AMLM5

ERROR=((Total-Calculated)/Total+1E-30)*100

MASSBBAL=Total-Calculated + 1

;================================================================================

;Calculation with model

;================================================================================

;calations for graphs (Figure 6)

PercHE = (AMLHE)*100/DOSE ;percentage of the dose metabolized to HE

PercAP = (AMLAP)*100/DOSE ;percentage of the dose metabolized to AP

PercEE = (AMLEE)*100/DOSE ;percentage of the dose metabolized to EE

PercHA = (AMLHA)*100/DOSE ;percentage of the dose metabolized to HA

PecM5 = (AMLM5)*100/DOSE ;percentage of the dose metabolized to M5

PercHES = (AMLHES)*100/DOSE ;percentage of the dose metabolized to HES

PercHEG = (AMLHEG)*100/DOSE ;percentage of the dose metabolized to HEG

PercOE = (AMLOE)*100/DOSE ;percentage of the dose metabolized OE
